# Magnetically Controllable and Degradable Milliscale Swimmers as Intraocular Drug Implants

**DOI:** 10.1002/advs.202507569

**Published:** 2025-06-17

**Authors:** Erdost Yildiz, Ugur Bozuyuk, Eray Yildiz, Fan Wang, Mertcan Han, Alp Can Karacakol, Devin Sheehan, Yan Yu, Metin Sitti

**Affiliations:** ^1^ Physical Intelligence Department Max Planck Institute for Intelligent Systems Stuttgart Germany; ^2^ Koç University Research Center for Translational Medicine (KUTTAM) Koç University Istanbul Turkey; ^3^ Department of Molecular Biology and Genetics Izmir Institute of Technology Izmir Türkiye; ^4^ Institute for Biomedical Engineering ETH Zurich Zurich Switzerland; ^5^ School of Medicine and College of Engineering Koç University Istanbul Türkiye

**Keywords:** biodegradation, hydrogel, intraocular drug implants, microrobotics, retinal diseases

## Abstract

Intraocular drug implants are increasingly used for retinal treatments, such as age‐related macular degeneration and diabetic macular edema, due to the rapidly aging global population. Although these therapies show promise in arresting disease progression and improving vision, intraocular implant‐based therapies can cause unexpected complications that require further surgery due to implant dislocation or uncontrolled drug release. These frequent complications of intraocular drug implants can be overcome using magnetically controllable degradable milliscale swimmers (MDMS) with a double‐helix body morphology. A biodegradable hydrogel, polyethylene glycol diacrylate, is employed as the primary 3D printing material of MDMS, and it is magnetized by decorating it with biocompatible polydopamine‐encapsulated iron‐platinum nanoparticles. MDMS have comparable dimensions to commercial intraocular implants that achieve translational motions in both aqueous and vitreous bodies. They can be imaged in real‐time using optical coherence tomography, ultrasound, and photoacoustic imaging. Thanks to their biodegradable hydrogel‐based structure, they can be loaded with anti‐inflammatory drug molecules and release the medications without disrupting retinal epithelial viability and barrier function, and decrease proinflammatory cytokine release significantly. These magnetically controllable swimmers, which degrade in a couple of months, can be used for less invasive and more precise intraocular drug delivery compared to commercial intraocular drug implants.

## Graphical abstract

Magnetically controllable degradable milliscale swimmers (MDMS) offer a promising alternative to commercial intraocular drug implants, reducing complications like implant dislocation and uncontrolled drug release. MDMS achieve high velocities in ocular environments, enable real‐time imaging, and deliver drugs effectively without disrupting retinal viability. They significantly reduce proinflammatory cytokine release, suggesting a safer, more reliable treatment for retinal diseases.

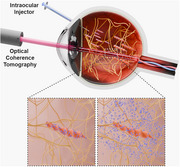

## 1 Introduction

Currently, the burden of retinal diseases, including diabetic retinopathy, age‐related macular degeneration, and uveitis, is increasing due to the aging population, affecting more than 400 million people worldwide, and is expected to rise to 600 million by 2040.^[^ ^1^, ^2^, ^3^, ^4^ ^]^ For the treatment of all these ocular disorders, intraocular drug implants are promising therapeutic tools in clinical settings, and it seems that their usage areas will become more and more widespread, but they are still in early stages in terms of design.^[^ ^5^, ^6^ ^]^ Their ability to bypass the retina‐blood barrier, site‐specific drug delivery capabilities, and long‐lasting, consistent drug release for medications with minimal side effects on other organs compared to systemic administration made them a crucial part of the clinical ophthalmology practice.^[^ ^7^ ^]^ Thanks to the current microfabrication and polymer technologies, we can build much more complex and smart drug carriers for the sustained release of pharmacological agents.^[^ ^8^ ^]^ Despite these developments, we face inefficient therapy and serious complications, such as implant displacement to the anterior chamber of the eye, in the current use of intraocular medical implants.^[^ ^9^, ^10^ ^]^ Untreatable retinal diseases, removal surgeries for displaced implants, and implant‐related infectious and inflammatory conditions on the healthy parts of the retina still affect ophthalmology clinical practice at a significant rate.^[^ ^11^ ^]^ Although intraocular drug implants are the first‐line treatment for many of these retinal diseases, thanks to their high bioavailability compared to other drug administration methods (topical or systemic),^[^ ^12^, ^13^ ^]^ complications related to these implants are seen frequently, in ≈2% of patients.^[^ ^14^ ^]^ Nondegradable intraocular implants, such as polyvinyl alcohol (PVA) based ones, may interfere with vision by migrating into the anterior chamber or visual field, whereas degradable intraocular implants, such as polylactic‐co‐glycolic acid (PLGA) based ones, may disrupt ocular physiology by causing inflammation or acidification within the eye due to degradation products.^[^ ^15^, ^16^, ^17^ ^]^ A detailed comparison of the intraocular drug implants can be found in Table S1 (Supporting Information). Even after an uncomplicated intraocular implantation procedure, the distribution of the drug in the posterior segment of the eye remains in a limited area due to the combination of nonspecific attachments, advective and diffusive transport mechanisms,^[^ ^18^, ^19^ ^]^ and a displaced implant, due to eye movements, which could not stop the progression of the retinal disease,^[^ ^20^ ^]^ may even worsen the patient's condition and necessitate removal surgery.^[^ ^21^, ^22^ ^]^ Considering other factors, such as the limited visibility of the implants under optical coherence tomography (OCT), which is the most commonly used imaging modality in ophthalmology, and the fact that currently commercially available intraocular implants can only be used with small molecule drugs, their pharmaceutical efficacy and duration remain insufficient for many patients.^[^ ^23^ ^]^ Additionally, the necessity of surgical intervention in the event of complications^[^ ^9^ ^]^ underscores the urgent need for innovative engineering solutions in intraocular drug delivery.^[^ ^24^ ^]^

In recent years, wireless small‐scale robotic devices have been increasingly tested in many clinical applications.^[^ ^8^ ^]^ However, due to many biomedical problems, including biocompatibility, foreign body response, and biodegradability, they still cannot be put into routine use in clinical settings.^[^ ^25^ ^]^ Although many state‐of‐the‐art microfabrication and chemical synthesis methods have been combined to build degradable magnetic robots that can carry pharmaceutical agents,^[^ ^26^ ^]^ realistic clinical scenarios, guidelines, and procedures should be planned in collaboration with clinicians in order to test these small‐sized robots on patients in the clinics.^[^ ^27^ ^]^ With the recent proliferation and affordability of microfabrication techniques, such as 3D digital light processing (DLP) bioprinting, these small robotic devices can be manufactured at high throughput for clinical applications, and the clinical translation process of these devices can become more accessible, faster, and inexpensive.^[^ ^28^, ^29^ ^]^

Even though many medical microrobotics research groups have tried to build millimetric or microscale robotic systems that can deliver therapeutic agents to the posterior chamber of the eye to treat aforementioned diseases, none of these devices have made it to viable clinical applications yet. Ulrich et al. claimed that microrobots could be used for ophthalmological surgical and pharmaceutical treatments, but due to the size and shape (cylinder with 1.8 mm length and 0.28 mm diameter) and the soft magnetic material of the milliscale robot in the study, it could not move appropriately in the vitreous humor, due to interweaved mesh structure of the vitreous,^[^ ^30^ ^]^ and had to be removed with the surgical operation it completed its task.^[^ ^31^ ^]^ When Wu et al. scaled down the size of the microrobots to pass through the mesh structure of vitreous humor,^[^ ^32^ ^]^ nondegradable nickel and perfluorocarbon containing, which are toxic to the retina,^[^ ^33^, ^34^, ^35^ ^]^ microrobots lost both their pharmaceutical carrier capabilities and surgical functionalities due to their small size.^[^ ^36^ ^]^ When we consider recent microrobotic literature together with the mentioned ophthalmology‐specific studies above, the volumetric drug transport capability limits in combination with controlled release problems and the difficulty of actuation in viscous biological media seem to be the most crucial physical problems to address in the transition to clinical usage.^[^ ^27^ ^]^ These problems can be solved by designing biocompatible magnetic nanoparticles‐decorated and hydrogel‐based small‐scale robots with physical intelligence in light of their targeted clinical functionality.^[^ ^37^, ^38^, ^39^ ^]^

**Figure 1:**
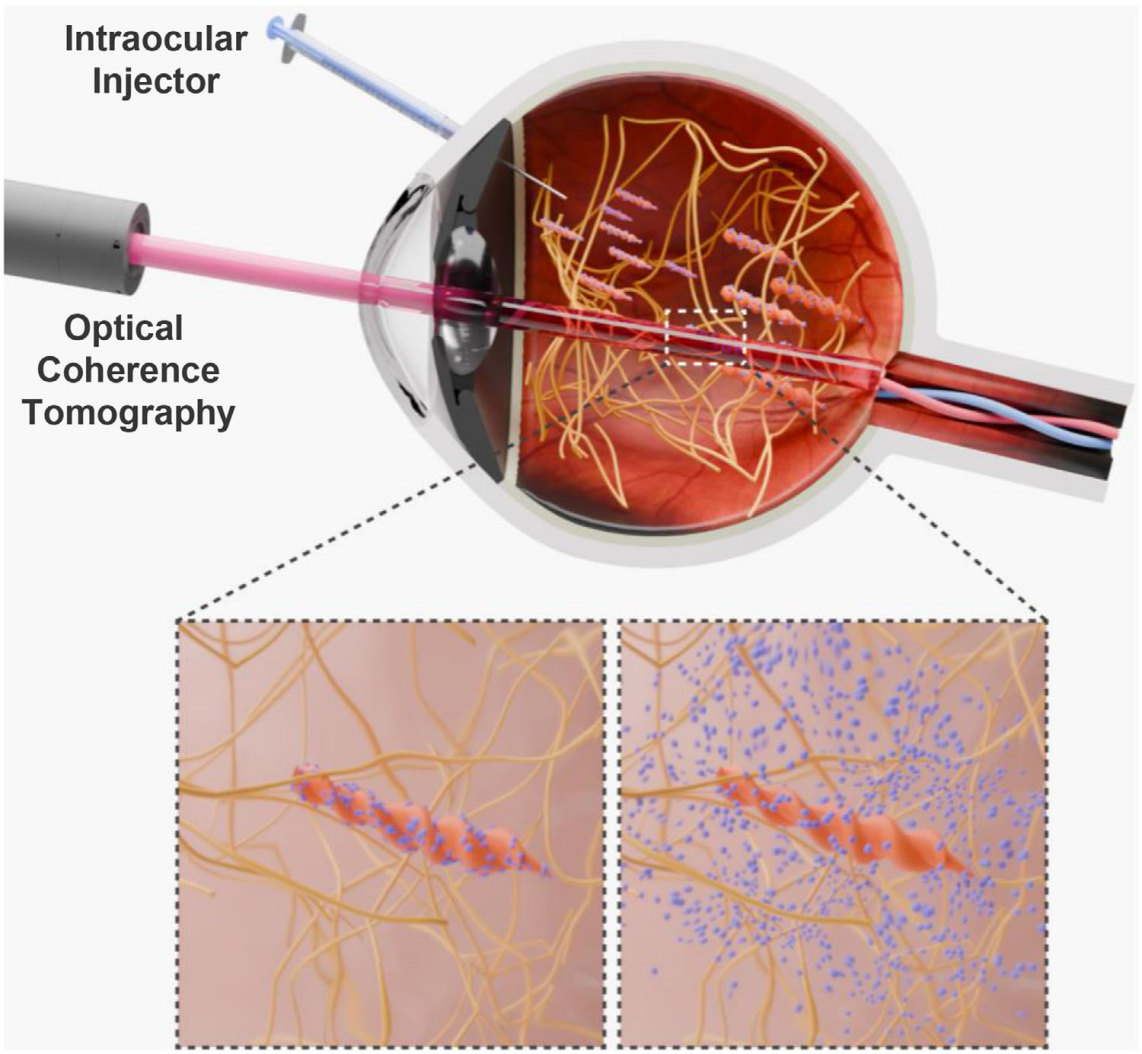
Schematics of the clinical scenario for the use of magnetically controllable degradable milliscale swimmers (MDMS) as intraocular drug implants. After the injection of MDMS with the guidance of optical coherence tomography (top), the MDMS drills inside the vitreous humor (bottom left) and releases the carried drug molecules passively by degradation (bottom right).

In this study, the limitations and complications of intraocular implants in the ophthalmology clinic are considered while trying to find solutions to these problems with recent developments in wireless small‐scale robotics.^[^ ^8^, ^40^ ^]^ While the double helical swimmer design was selected as the design with the highest drug carriage capability, compared to other helical swimmer designs, with a programmable magnetic actuation performance in viscous liquids,^[^ ^41^, ^42^ ^]^ in order to increase translational rotational movement inside the vitreous body, the amount and duration of the drug transported and released from the magnetic microscale robots were extended by building them in a design similar in size to commercial intraocular implants.^[^ ^43^ ^]^ Unlike other micro‐ or milliscale robot studies, a degradable hydrogel, polyethylene glycol diacrylate (PEGDA), was employed as the primary 3D bioprinting material of a small‐sized soft robot, leveraging its controllable degradation and drug immobilization features, and it was magnetized by decorating it with biocompatible and ferromagnetic iron platinum (FePt) nanoparticles.^[^ ^44^ ^]^ In addition to that, the milliscale robot coated with polydopamine (PDA), which reduces the possibility of the immune response,^[^ ^45^ ^]^ increases implant visibility during optical imaging methods and opens the possibilities of surface functionalization for milliscale robot customization for specific cell targeting in the posterior segment of the eye. This integrative and interdisciplinary approach (**Figure** **1**) enabled the development of 3D‐printed, magnetically controlled degradable milliscale swimmers (MDMS) as intraocular drug implants. In this way, MDMS address the intraocular implant dislocation‐related complications, enable more precise, medical imaging‐guided drug delivery to retinal lesions, and eliminate the need for additional surgeries to retrieve the implant after its mission is complete, thanks to their biodegradability.

## 2 Results

### 2.1 Design, Fabrication Process, and Material Characterization of MDMS

**Figure 2:**
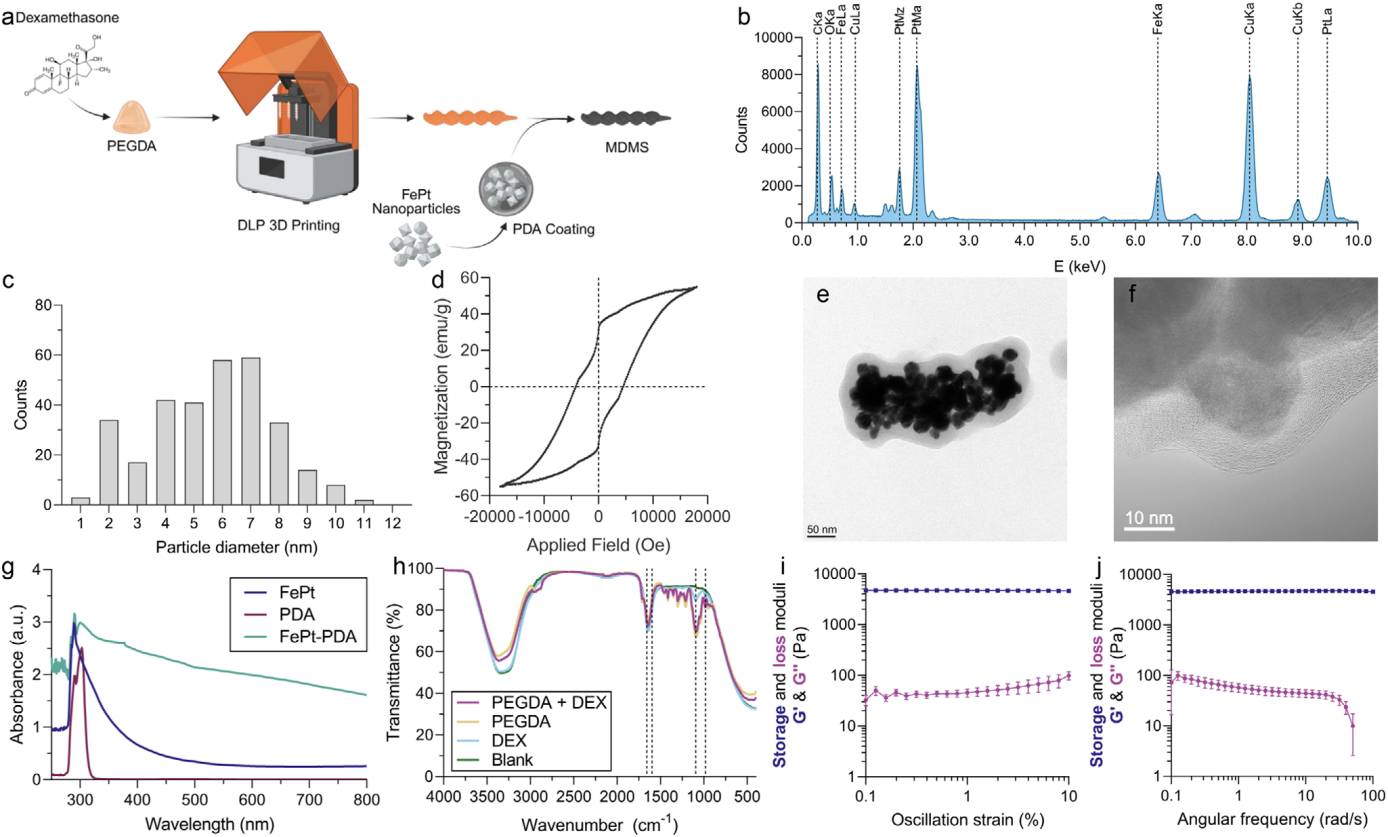
Fabrication and material characterization of MDMS and its ingredients. a) Production pipeline for MDMS. The carried drug dissolves in the PEGDA solution before 3D printing. DLP‐based 3D printing is used to produce double helical‐shaped swimmers. After the 3D printing, the MDMS were incubated in the PDA‐coated FePt nanoparticle solution to cover them with a ferromagnetic shell. b) EDX analysis results for FePt nanoparticles with corresponding peaks for the specific elements. While Fe and Pt peaks are measured from nanoparticles, Cu and C peaks come from the SEM measurement grid. c) The particle diameter distribution of FePt nanoparticles. d) The magnetic hysteresis curve of the FePt nanoparticles indicates hard ferromagnetic characteristics. e) The TEM image of the PDA coating of FePt nanoparticles. The scale bar is 50 nm. f) STEM image of the PDA coating of the nanoparticles indicates a 7.79 ± 1.56 nm thickness PDA coating. The scale bar is 10 nm. g) Ultraviolet (UV)‐Visible light spectroscopy results for FePt, PDA precursor, and PDA‐coated FePt nanoparticle solutions. h) Fourier transform infrared (FTIR) spectroscopy results for blank and DEX‐loaded PEGDA hydrogel precursors. The dotted lines indicate the corresponding peaks for DEX. i) The storage and loss moduli of the MDMS under varying oscillation strain (%). j) The storage and loss moduli of the MDMS under varying angular frequency (rad/s).

The production process consists of three main straightforward steps: dexamethasone loading to the 3D printing pre‐mix, digital light processing‐based 3D printing of double helical milliscale swimmers, and coating the swimmers with PDA‐encapsulated FePt nanoparticles (**Figure** **2**a). By combining the actuation capabilities of double helical swimmers in the low Reynolds number regime with biodegradable hydrogels and magnetic nanoparticles,^[^ ^26^, ^46^ ^]^ we achieved a unique small‐scale robotic design with a soft degradable drug carrier body and a ferromagnetic hard shell for actuating the double helical swimmer, which can carry ≈3.29 mg of dexamethasone in each swimmer, which is over four times higher than the commercial counterparts, including Ozurdex (Table S1, Supporting Information). The MDMS's glucocorticoid loading capacity is achieved by dissolving dexamethasone phosphate in the 3D printing precursor solution. While the compounds of the 3D printing pre‐mix were purchased from commercial producers, ferromagnetic FePt nanoparticles with PDA coating were produced in‐house for controllable particle size and shape, stability, and biocompatibility. FePt nanoparticles, produced via one‐pot single‐step synthesis,^[^ ^47^ ^]^ have significantly higher magnetic hysteresis and biocompatibility than the other ferromagnetic materials.^[^ ^48^ ^]^ According to energy dispersive X‐ray spectroscopy (EDX) measurements, these FePt nanoparticles consist of 42.72 ± 0.93% iron and 57.29 ± 0.93% platinum (Figure 2b; Figure S1b–d, Supporting Information), and the mean diameter of the FePt nanoparticles is 5.62 ± 2.14 nm (Figure 2c). The magnetization of the FePt nanoparticles was measured between ‐55 and 55 emu g^−1^ in an applied magnetic field from ‐18000 to 18000 Oe (Figure 2d), which proves its high potential to be employed as the magnetic coating material.

The FePt nanoparticles were coated with PDA with overnight shaking to create a FePt nanoparticle suspension in physiological media, which is phosphate‐buffered saline (PBS). In the super‐resolution TEM images, the mean thickness of the PDA coating of the nanoparticles was measured as 7.79 ± 1.56 nm, and aggregations of the FePt nanoparticles were observed (Figure 2e,f; Figures S1a and S2, Supporting Information). In the macroscopic scale, the change in the color and absorption spectra of the PDA‐coated FePt solution was measured via UV‐Visible spectrophotometer. Due to the polymerization of dopamine hydrochloride to the polydopamine chains,^[^ ^49^ ^]^ the optical absorption spectrum of the solution widened up to 800 nm (Figure 2g).

While biocompatible ferromagnetic nanoparticles (FePt@PDA) were integrated into the magnetic shell of the controllable implant, drug loading into the PEGDA‐based DLP 3D printing pre‐mix was quantified using FTIR spectroscopy, leveraging the distinct optical signature of dexamethasone in the infrared spectrum (Figure 2h; Figure S3, Supporting Information). The peaks of dexamethasone in FTIR spectroscopy were observed in 982, 1094, 1598, and 1658 cm^−1^. Compared to the other hydrogel precursors, PEGDA was selected for the DLP‐based 3D printing of the MDMS thanks to its comparable drug‐loading capacity, stiffness (Table S2, Supporting Information), and lower immunoreactivity compared to PLGA‐based implants.^[^ ^17^, ^50^ ^]^ When the 3D printing and FePt@PDA coating were completed for the MDMS, its storage moduli showed consistent steady‐state values in the range of 4.68 ± 0.07 and 4.70 ± 0.03 kPa in frequency and amplitude sweep modes, respectively, which indicates stable viscoelastic hydrogel behavior of the MDMS (Figure 2i,j). Because the mechanical strength of the MDMS are in the sweet spot between the vitreous,^[^ ^51^ ^]^ which is 2.8 ± 0.9 Pa, and the retina,^[^ ^52^ ^]^ which is 18.6 ± 16.3 kPa, it can traverse through the vitreous without any risk of mechanical damage (e.g., penetration) to the retina. Following material characterizations, MDMS with a length of 6 mm and a radius of 500 µm (Figure S4, Supporting Information) were magnetized perpendicularly to their long axis under a uniform 1.8 T magnetic field with a vibrating sample magnetometer (VSM) and subsequently employed in actuation experiments.

### 2.2 Deployment, Actuation, and Medical Imaging of MDMS

**Figure 3:**
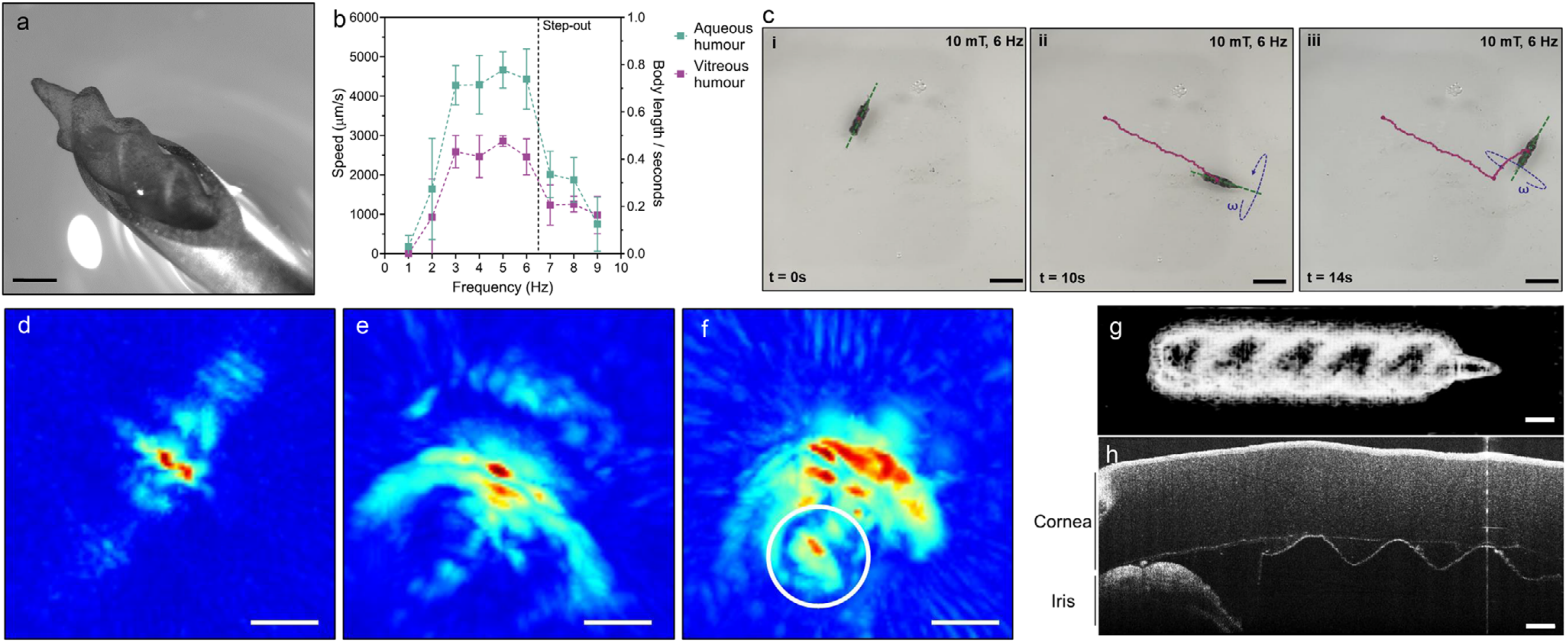
Magnetic actuation, photoacoustic imaging, and OCT‐based medical imaging of MDMS. a) Photo of the MDMS in the 17G needle. The scale bar is 500 µm. b) The velocity of the MDMS under various frequencies of the rotating magnetic field (n=6). The MDMS steps out after a 6.5 Hz rotating magnetic field speed. c) Snapshots of MDMS's controlled swimming trajectory (purple) in the vitreous humor, with the rotation direction (blue) and axis (green) of the magnetic field. The scale bar is 5 mm. d) Photoacoustic image of the MDMS in the agar. e) Photoacoustic image of the porcine eye before injection of MDMS. f) Photoacoustic image of the same porcine eye after the injection of MDMS. The location of MDMS is indicated with a white circle. The scale bars in the photoacoustic images are 10 mm. g) En‐face OCT image of the MDMS. The scale bar is 500 µm. h) The sectional OCT image of the MDMS in the porcine eye. The outline of the double‐helical swimmers can be seen alongside the corneal endothelium. The scale bar is 500 µm.

While it is rarely mentioned in the literature, the deployment of micro‐ and milliscale robots is a significant challenge for biomedical microrobotic studies.^[^ ^53^ ^]^ We built the MDMS, which can be easily delivered with 17G needles (**Figure** **3**a). Even though 17G needles are rarely used in ophthalmology, they create significantly less ejection force and smaller incision sizes compared to commonly used intraocular injectors, such as Accuject.^[^ ^54^ ^]^ After the injection, MDMS can move in both the aqueous and vitreous bodiesunder the rotating magnetic fields generated by a rotating permanent magnet. The large working distance and vast programmability of the rotating permanent magnet enable precise control of MDMS on a parallel plane to the rotating permanent magnet. MDMS act in master‐slave coupling with the permanent magnet, rotating and aligning its direction accordingly (Figure S5a, Supporting Information). The rotation frequency of the magnetic field was increased from 1 to 9 Hz to investigate the step‐out frequency. The MDMS can be steered and actuated at 6 Hz in both aqueous and vitreous humor (Figure 3b). The MDMS achieved velocities of 4.28 ± 0.50 mm s^−1^ (0.71 ± 0.08 blps) and 2.59 ± 0.41 mm s^−1^ (0.43 ± 0.06 blps) at 3 Hz, and their speed stayed constant at 4.43 ± 0.76 mm s^−1^ (0.74 ± 0.13 blps) and 2.46 ± 0.46 mm s^−1^ (0.41 ± 0.08 blps) until 6 Hz, respectively in the aqueous and vitreous humor. Above 6 Hz, the MDMS started to step out and did not achieve the rotation frequency of the magnetic fields for helical swimming. Its unique double‐helical swimmer configuration allows the MDMS to move forward and navigate in both aqueous and vitreous media (see Figure 3c; Figure S5b and Video S1, Supporting Information). Its movement is better described as helical drilling rather than helical swimming, given that it drills into the fibrillary mesh of the vitreous humor. This is the first small‐scale biodegradable soft robot that can actuate inside a mesh‐like extracellular matrix of a mammalian eye, which is the vitreous humor in this study.

Beyond magnetic actuation, the MDMS were visualized using three different biomedical imaging modalities: photoacoustic (PA), OCT, and ultrasound (US) imaging. While we utilized PA as an emerging imaging modality, we incorporated OCT and US in our study because these are the modalities employed in nearly all ophthalmology clinics.^[^ ^55^, ^56^ ^]^ Also, our group previously demonstrated FePt's functionality as a contrast agent in PA imaging.^[^ ^39^ ^]^ First, we observed MDMS in the ex vivo porcine eye using PA imaging. The MDMS were initially imaged within agar, then we injected it into the posterior chamber of the porcine eye with a sterile 17G injector. MDMS can be seen as a helical structure under photoacoustic imaging due to the IR spectral absorbance of PDA‐coated FePt particles on its surface (Figure 3d). The same helical structure can be identified in the porcine eye after injection (Figure S6 and Video S2, Supporting Information). To demonstrate MDMS's visibility under PA imaging, we imaged the same porcine eye before and after the injection (Figure 3e,f). Lastly, the helical swimming of the MDMS were observed with a 3D photoacoustic imaging transducer (Video S3, Supporting Information).

When we image the MDMS in both the intraocular bodies and ex‐vivo porcine eye with OCT, we were able to see the hyperreflective borders of the swimmer in the B‐scan sectional images in both stationary and moving conditions (Figure 3g,h; and Video S4, Supporting Information). After we collected and combined the B‐scan images, we were able to enhance the MDMS's visibility by reconstructing the en‐face OCT projection (Figure 3g). Intraocular localization of the MDMS can be determined with the OCT, and it can be moved to the desired locations for drug release (Figure 3h). Although the MDMS can be trackable in coronal sections during its translational movement under OCT (Video S4, Supporting Information), the 3D reconstruction of the moving implant is not possible, due to the slow acquisition speed of the interferometry‐based OCT technique. In addition to the PA and OCT imaging modalities, we were also able to image the MDMS under US imaging (Video S5, Supporting Information). While the 3D structure can be seen in the PA imaging, only sections of the MDMS can be seen in OCT and US, due to the sectional real‐time imaging. Leveraging the near‐infrared emission and hyperechoic feature of the PDA‐coated FePt nanoparticles, the actuation of the MDMS can be tracked with PA, OCT, or US imaging after their injection into the eye, which enables clinicians to track the location of the MDMS inside the eye during the treatment period.

### 2.3 Drug Delivery and Biodegradation of MDMS

**Figure 4:**
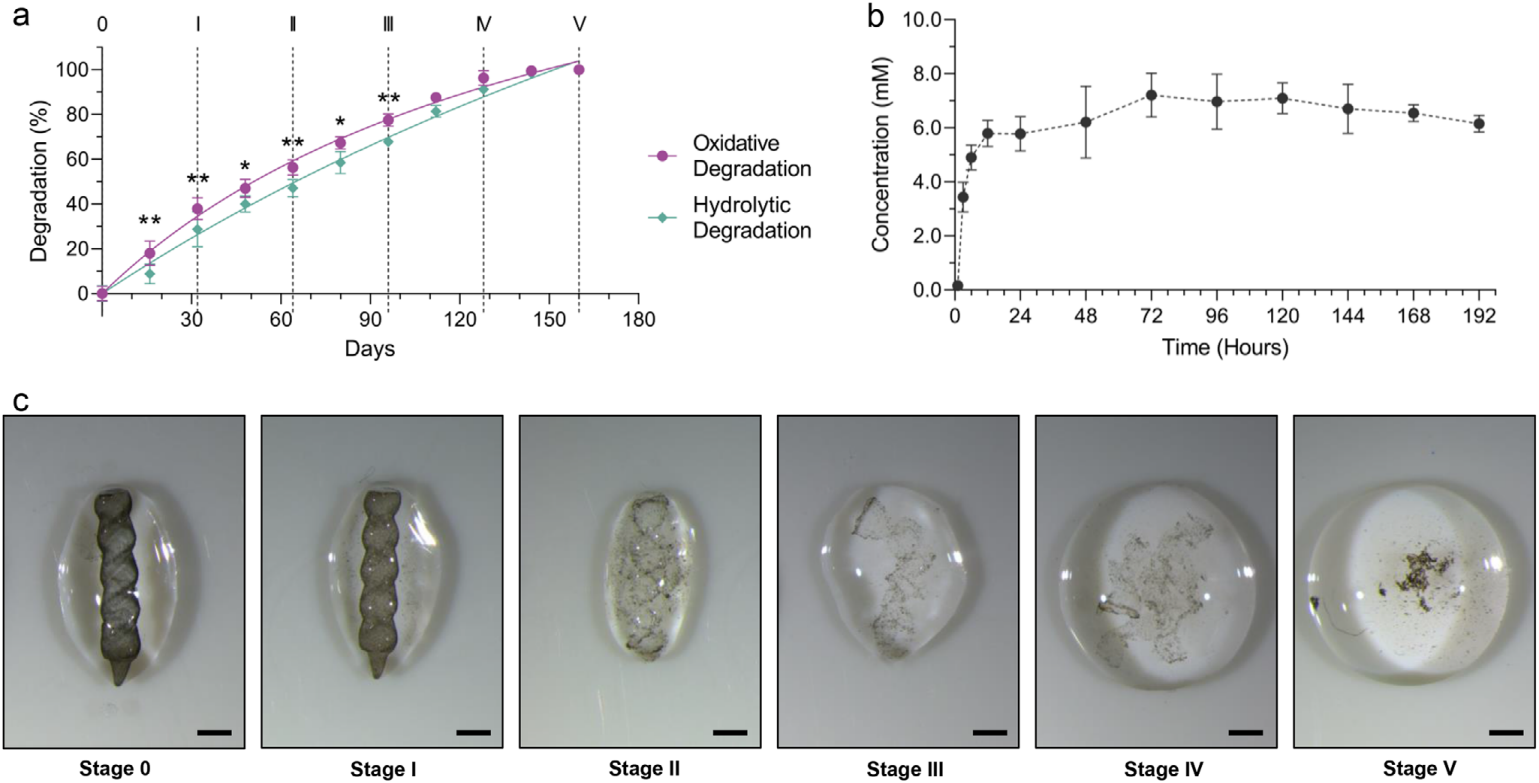
Degradation and drug release properties of MDMS. a) Percentage of the degradation of MDMS by time in the oxidative and hydrolytic degradation conditions. * indicates *p* < 0.05 and ** indicates *p* < 0.01. b) The DEX concentrations by time in the cell culture media with the MDMS are measured with high‐performance liquid chromatography. c) The snapshots of the stages of the hydrolytic degradation of the MDMS. The number of stages corresponds to the numbered time points in the degradation plot. The scale bar is 1 mm.

As a next step in the study, we investigated its degradation mechanisms under accelerated aging conditions and drug release mechanisms. We conducted two different accelerated aging tests, hydrolytic and oxidative degradation conditions, to understand the mechanisms of degradation for the MDMS. While we used hydrogen peroxide solution for the accelerated oxidative degradation, we used sodium peroxide solution for the accelerated hydrolytic degradation. We observed that oxidative stress increases the degradation of the MDMS more than hydrolytic stress (**Figure** **4**a). While the MDMS degrade completely after 18 days under accelerated oxidative degradation conditions, which corresponds to 144 days in in vivo conditions,^[^ ^57^ ^]^ and it degrades completely at day 20 under accelerated hydrolytic degradation conditions, which corresponds to 160 days in normal conditions inside the biological tissue. When we examined the hydrolytic degradation stages, we found that the dexamethasone‐loaded PEGDA body completely lost its shape and disappeared over time, leaving only iron platinum nanoparticle aggregates (Figure 4c). In parallel with the biodegradation experiments, we examined the amount of drug released from MDMS under in vitro conditions in ARPE‐19 retinal pigment epithelium cell culture. For this purpose, we incubated the MDMS samples in the DMEM/F12 medium on the ARPE‐19 cell layer and measured the released dexamethasone amounts with high‐pressure liquid chromatography (HPLC) in the cell culture conditions (Figure S7, Supporting Information). The MDMS demonstrated a zero‐order drug release kinetics, which is favorable for the long‐term consistent drug release from the implants (Figure 4b).^[^ ^58^ ^]^ The zero‐order drug release kinetics is achieved with a core‐shell structure by combining PDA‐based coating with a PEGDA‐based soft hydrogel body for a small molecular‐sized drug, dexamethasone.^[^ ^59^ ^]^ The released dexamethasone concentration reaches 6.21 ± 2.30 mm at 48 h and remains at a similar level, 6.16 ± 0.53, through 192 h, significantly higher than the maximum dexamethasone concentration of commercial implants, which is 0.2 ± 0.93 mm.^[^ ^60^ ^]^

### 2.4 Biocompatibility, Immunocompatibility, and Therapeutic Effects of MDMS

**Figure 5:**
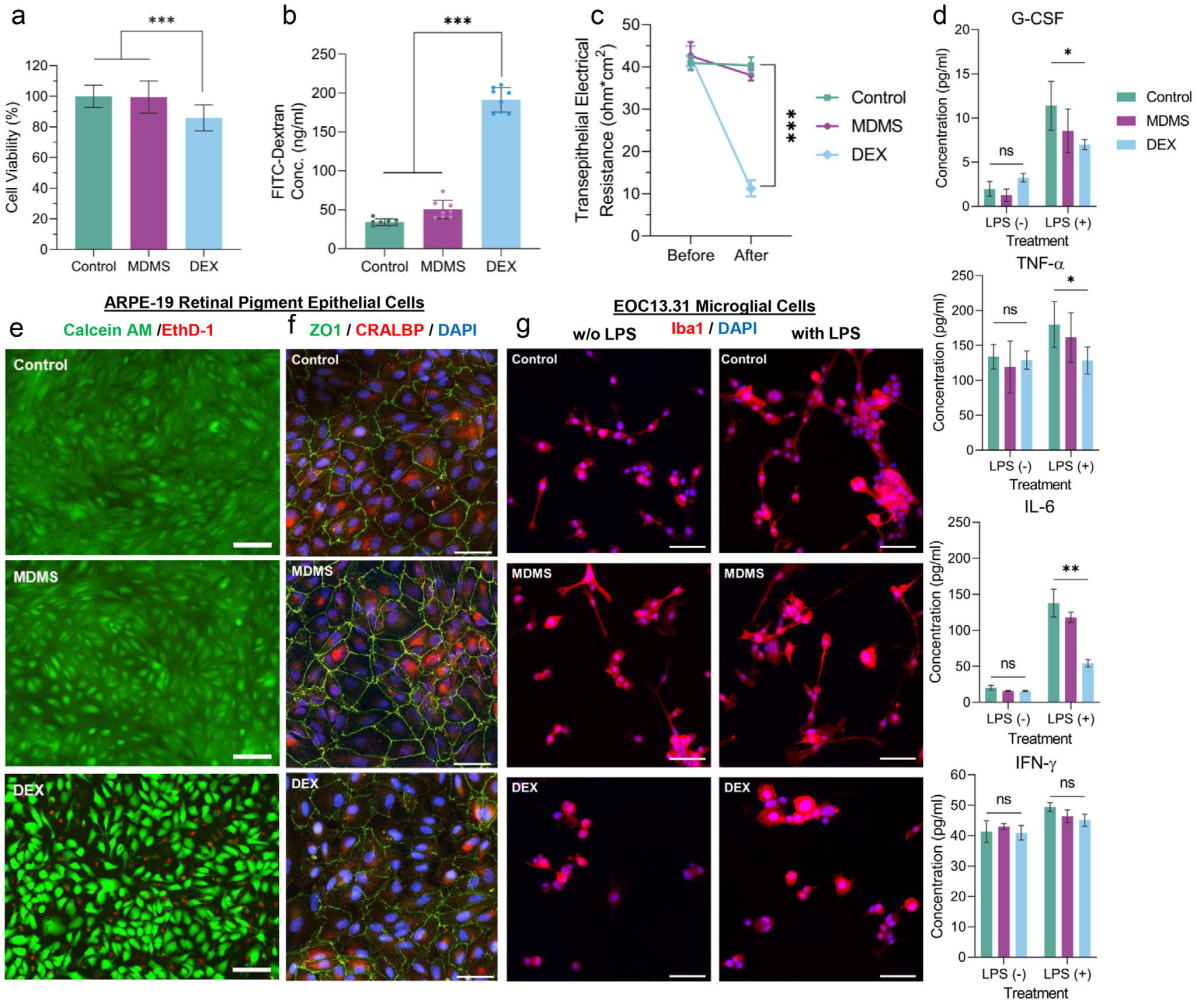
Effects of MDMS on retinal pigment epithelium and microglia under normal and inflammatory conditions. a) The percentages of viable ARPE‐19 retinal pigment epithelium cells in the nontreated, MDMS‐, and DEX‐treated groups (n = 16). b) FITC‐dextran‐based transwell membrane permeability test results with ARPE‐19 cell layers for nontreated, MDMS‐, and DEX‐treated groups (ng/ml) (n = 8). c) Transepithelial electrical resistance (ohm×cm^2^) results of ARPE‐19 cell layers on transwell membrane for nontreated, MDMS‐, and DEX‐treated groups, before and after treatment (n = 4). d) ELISA measurements of G‐CSF, TNF‐α, IL‐6, and IFN‐γ concentrations in the EOC13.31 microglial cell media for nontreated, MDMS‐, and DEX‐treated groups with and without LPS treatment (n = 4). e) Live cell fluorescence images of the ARPE‐19 cells under control, MDMS, and DEX treatments. While green fluorescence indicates Calcein AM, a live cell indicator, red fluorescence indicates Ethidium Homodimer‐1 (EthD‐1), a dead cell indicator. The scale bar is 100 µm. f) Immunofluorescence images of the ARPE‐19 cells under control, MDMS, and DEX treatments. The green fluorescence indicates zona occludens 1 (ZO‐1) proteins, which constitute the tight junctions; red fluorescence indicates CRALBP proteins, which are ARPE‐19 cell‐specific proteins, and blue fluorescence indicates DAPI, a nuclear marker. The scale bar is 100 µm. g) Immunofluorescence images of the EOC13.31 cells under control, MDMS, and DEX treatments with or without LPS. While red fluorescence indicates Iba‐1, which is a microglia‐specific structural protein, blue fluorescence indicates DAPI, a nuclear marker. The scale bar is 100 µm. “ns” indicates *p* ≥ 0.05, * indicates *p* < 0.05, ** indicates *p* < 0.01, and *** indicates p<0.001, the p‐value range for the statistical difference compared to the control group.

For biocompatibility and immunocompatibility studies of the MDMS, we used ARPE‐19 cells as a cell culture model for retinal pigment epithelium and EOC13.31 cells as a microglial cell model, respectively.^[^ ^61^, ^62^ ^]^ In addition to the cytotoxicity tests for the MDMS, its effects on epithelial barrier function were also investigated both functionally and morphologically. The ARPE‐19 retinal epithelial cells treated with MDMS showed significantly higher viability after 48‐h incubation, compared to the equivalent dexamethasone amount directly given to the cell culture media (*p* < 0.001, 99.50% ± 10.58% vs. 85.82% ± 8.49%, respectively) (**Figure** **5**a,e). Also, the slow dexamethasone release profile of the MDMS help the ARPE‐19 cell layer to protect its epithelial barrier function. While we can observe decreased tight junction formations and intercellular leakage points on the only dexamethasone‐added ARPE‐19 cells, the ARPE‐19 cells with the MDMS show similar morphological properties to the control group in immunofluorescence images (Figure 5f). Protection of epithelial barrier function in ARPE‐19 cells incubated with the MDMS is also shown in functional transwell membrane assays. When the FITC‐tagged dextran with 40 kDa molecular weight (FITC‐dextran) was added to the upper chamber of the transwell, its passage to the lower chamber was significantly higher in the dexamethasone group, while FITC‐dextran passage was comparably low in the control and the MDMS groups (*p* < 0.001, 191.30 ± 15.69, 34.20 ± 4.28, and 50.47 ± 11.74 ng ml^−1^, respectively) (Figure 5b). In addition to FITC‐dextran transwell membrane permeability tests, we measured transepithelial electrical resistance (TEER) values before and after treatments of the ARPE‐19 cell layers on transwell membranes. While the TEER values for the ARPE‐19 cells stayed the same before and after the incubation period in control and MDMS groups, the TEER level significantly decreased after dexamethasone incubation (*p* < 0.001, before and after TEER difference are 4.565 ohm cm^2^ for MDMS and 31.32 ohm cm^2^ for dexamethasone groups) (Figure 5c). All these results indicate that MDMS can carry and achieve sustained release of dexamethasone, without disrupting epithelial barrier function on retinal pigment epithelium.

As the last step of the in vitro investigation of MDMS, we observed the immune response of EOC13.31 microglial cells to the MDMSs. The morphology and interleukin release of the microglia are observed with and without bacterial lipopolysaccharide (LPS, 2 µg mL^−1^) incubation. The microglia showed significantly lower inflammatory cytokine response compared to only LPS administration when we added the MDMS into the LPS‐supplemented cell culture media (Figure 5d). The inflammation‐related cytokines, G‐CSF, TNF‐α, and IL‐6 concentrations significantly lowered with MDMS administration to LPS in a similar fashion to bolus dexamethasone administration. These ELISA results for inflammation‐related cytokines indicate a comparable therapeutic capability of MDMS with bolus dexamethasone administration. When we observed the microglia under a fluorescence microscope, the microglial morphology and survival were not affected negatively by dexamethasone‐loaded MDMS with or without LPS presence, contrary to microglia in the 48 h incubation after the bolus dexamethasone injection (Figure 5g). The MDMS have protected microglial cells while achieving inflammation suppression capability on microglial cells, which are the primary regulators of the immune response in the retina.

## 3 Discussion

In this study, we redesigned the intraocular drug implant with physical intelligence, which combines the physical properties of the structural materials of the device to create an intelligent holistic design, to address pressing problems in retinal medical therapies, and we developed a sustained drug release implant for dexamethasone with actuation capability in the intraocular fluids thanks to 3D hydrogel printing and programmable magnetic nanoparticles. We built the MDMS with a soft hydrogel body that carries the drug molecules, coated with a magnetic shell, which enabled us to drive it with magnetic fields under medical imaging guidance. This PEGDA‐based implant design led to a slow release profile after initial fast release.^[^ ^58^, ^59^ ^]^ The MDMS implant achieved therapeutic drug loading and delivery capacities, around four times higher than commercial equivalents (Table S1, Supporting Information), untethered controllability with the combination of external magnetic fields and medical imaging modalities, and significant biocompatibility and therapeutic efficiency regarding epithelial barrier and immune response. Compared to the current commercial dexamethasone implant, such as Ozurdex, the MDMS deliver more than four times the drug in a single injection, 0.7 mg versus 3.29 mg, and is easier to visualize under interferometric and acoustic imaging modalities compared to commercial implants.^[^ ^63^, ^64^ ^]^ While the MDMS consisted of a therapeutic amount of glucocorticoid, which decreased the pro‐inflammatory interleukin release on microglia, we did not observe acute complications of the bolus glucocorticoid administration on the retinal pigment epithelium, such as disruption of the epithelial barrier function, thanks to its slow drug release profile.^[^ ^65^ ^]^ In contrast to previous commercial intraocular implants, the anatomical location of the MDMS could be determined with ultrasound‐based techniques, including US and PA, and interferometry‐based biomedical imaging techniques, including OCT, which is one of the most frequently used medical imaging modalities in ophthalmology, thanks to its unique helical shape and magnetic nanoparticle‐based coating. In addition to these features, it is the first biodegradable hydrogel‐based small‐scale robot for treating ocular diseases, to the best of our knowledge.

While several hydrogel‐based magnetic small‐scale soft robots are used on different organ systems, this study was the first to investigate hydrogel‐based small‐scale robots for ophthalmologic applications.^[^ ^66^, ^67^ ^]^ Despite the numerous recent studies on polymer and graphene‐based magnetic microrobots, they are unable to move in viscous biological media, such as the vitreous body.^[^ ^68^, ^69^, ^70^ ^]^ In previous works on intraocular small‐scale robots, the researchers focused on the metal microrobots, which could actuate inside ocular fluids.^[^ ^31^, ^36^, ^71^ ^]^ Conversely, none of these robots have additional functionality and they cannot be used in a realistic clinical scenario, due to the toxicity of their structural components and very limited drug delivery capabilities. Also, due to their metal structures, the removal of the small‐scale robots was required after the implantation. In this study, we built a realistic small‐scale flexible and degradable robot with similar dimensions, 6 to 1 mm, to commercial intraocular implants (Table S2, Supporting Information), to deliver a realistic amount of drug, and to use commercial injectors in clinical settings. Although there is a lot of promise in the microrobotics literature, unfortunately, it is not realistic to achieve precise injection, medical imaging‐guided actuation, and long‐term sustained drug release with microrobots due to the physical limitations of the microscale structures.^[^ ^23^, ^27^ ^]^ Due to these reasons, we engineered the hydrogel‐based robot on the millimetric scale with a soft hydrogel core and hard magnetic shell to achieve long‐term zero‐order drug release. Thanks to the hard FePt and PDA coating on the outside and dexamethasone‐loaded PEGDA on the inside, MDMS act as a drug release chamber due to the osmotic behavior of the hydrogel body, providing zero‐order drug release, reducing drug‐dependent complications while still showing drug efficacy.^[^ ^58^ ^]^ This osmotic pump‐like structure of the MDMS enable fast swelling‐controlled drug transport in the acute period and slow erosion‐controlled drug release in the chronic period, which is proportionally linear with degradation.^[^ ^59^ ^]^ Compared to previous microrobot examples,^[^ ^36^ ^]^ which consist of toxic materials to the retina, such as nickel and perfluorocarbon, the MDMS are built with entirely biocompatible and biodegradable components, including polydopamine, PEGDA, and FePt, which enable it to degrade in the eye without any adverse effect or complications and their end products can be safely excreted from the body according to previous literature.^[^ ^50^, ^72^ ^]^ Even though there are residual FePt nanoparticles, which are far below the toxic amount,^[^ ^73^ ^]^ they will be excreted via the anterior, trabecular meshwork, or posterior transcranial routes with the help of retinal microglia without any complications.^[^ ^74^ ^]^ Thanks to DLP‐based 3D printing, it also proposes a straightforward, high‐throughput, and widely used fabrication method, compared to other commonly used small‐scale fabrication methods, such as two‐photon polymerization (Table S3, Supporting Information).^[^ ^38^, ^75^, ^76^ ^]^

As the most significant contribution of the study, the 3D‐printed magnetically controlled degradable milliscale swimmers can navigate within the vitreous humor while carrying significantly larger amounts of drugs than commercial equivalents, ≈3.29 mg of dexamethasone in this study. Their magnetically programmed nanoparticle‐based shells enable us to control them in the viscous environment more precisely than other types of small‐scale robots, such as optically or acoustically actuatable ones.^[^ ^77^, ^78^ ^]^ This magnetic control gives a unique advantage for the management of implant displacement‐related complications of intraocular devices. While complicated intraocular implants require complex and risky surgical operations to remove the implant,^[^ ^9^ ^]^ this magnetically controllable intraocular robot can be moved by a surgeon without any invasive procedure. In addition to magnetic steering, their magnetic nanoparticle coating helps with medical imaging as a contrast agent in photoacoustic, ultrasound imaging, and optical coherence tomography, which is readily available in most ophthalmology clinics. The slow, sustained release profile of the MDMS achieved therapeutic anti‐inflammatory effects on microglial cells without affecting the barrier integrity and viability of the retinal pigment epithelial cells,^[^ ^65^ ^]^ which could help to prevent clinical complications of intraocular glucocorticoid injections, including macular holes and elevated intraocular pressure.^[^ ^79^, ^80^ ^]^ In a similar fashion, MDMS decreased the inflammatory cytokine release from microglia without creating any negative effect on their viability. With all of the above‐mentioned features, MDMS provide solutions to common implant complications and inadequate medical treatment of retinal disease in the ophthalmology clinic with intelligent robotic design and innovative materials.

The current study has some limitations that can be improved in the future. In this study, our primary focus was on the design of magnetically controllable and degradable milliscale swimmers and the elucidation of their physical, chemical, and biological interactions in in vitro retina models. Consequently, experiments on animal models for eye diseases, such as New Zealand white rabbits or Ossabaw pigs, were not part of our experimental design, and we intend to collaborate with a clinical center and a large animal experimentation facility to compare the MDMS with its clinical equivalents in in vivo conditions for future studies. The dimensions of the MDMS approximate those of commercially available dexamethasone implants; however, the MDMS have the potential for miniaturization, which could minimize tissue trauma associated with intraocular injectors. The volumetric parameters of the microswimmer were precisely calculated to ensure long‐term, around six months, sustained therapeutic dexamethasone release following a single administration.^[^ ^65^ ^]^ Given the inherent limitations of milliscale double helical swimmers, a length‐to‐diameter ratio of 6:1 was determined to be optimal. This selection addresses two critical engineering challenges: elongated, thinner helical swimmers exhibit susceptibility to structural deformation, while shorter, thicker variants demonstrate compromised directional control due to tumbling phenomena.^[^ ^46^, ^81^ ^]^ Also, dexamethasone is used as a model drug in this study, even though it has some adverse effects on retinal homeostasis,^[^ ^82^, ^83^ ^]^ which does not exclude loading of other ophthalmic medications, especially antibody‐based ones, to the PEGDA‐based body of the MDMS.^[^ ^84^ ^]^ In concluding this discussion, a salient limitation is that drug release and biodegradation are not coupled with an external stimulus.

In future studies, we intend to engineer the MDMS to exhibit externally controllable degradability and drug release, incorporating an injection system that causes minimal tissue damage. We also plan to investigate the pharmacokinetics of various medications for their delivery with hydrogel‐based small‐scale robots to expand clinicians’ inventory for intravitreal robotic implants. Our studies will focus on the light‐controllable drug release mechanism for small‐scale hydrogel‐based magnetic robots. Different types of photocrosslinkers could help us release different medications in various visible light wavelengths.^[^ ^85^ ^]^ The combination of multi‐material 3D printing with digital light processing enables the loading of other drugs into small‐scale hydrogel‐based robots, which could reduce the number of intraocular injections.^[^ ^86^ ^]^ In addition to the drugs, the loading of photovoltaic or piezoelectric nanoparticles can be achieved with this cross‐linked PEGDA structure of the MDMS.^[^ ^87^, ^88^ ^]^ Lastly, the magnetic control system of the MDMS can be coupled with ocular imaging methods, especially OCT, to target multiple lesions and follow the therapeutic outcomes precisely.

## 4 Conclusion 

In this article, we tried to solve one of the most important problems in ophthalmology clinics, intraocular implant‐related complications, with a wireless millirobot design by leveraging the use of innovative materials and fabrication strategies. We rationally designed and engineered a small‐scale helical robotic device that combines what we have learned from the patient's bedside to the laboratory bench with inspiration from the movements of microorganisms in nature. The 3D‐printed hydrogel‐based magnetic miniature robotic design enables active intraocular implants with therapeutically effective drug doses that are externally controllable with magnetic fields, can be steered in the vitreous fluid, can release drugs continuously for long periods, are biocompatible, and are biodegradable. While this study is limited to the in vitro and ex vivo demonstration of the MDMS, it is the first small‐scale degradable robotic design for the treatment of retinal diseases. These studies would bring clinicians one step closer to using wireless robotic implants daily, thanks to the increased availability and affordability of novel robotic manufacturing methods.

## 5 Experimental Section

### Synthesis of PDA‐Coated FePt Nanoparticles

Briefly, 0.2 mM Platinum(II) acetylacetonate (282782, Sigma‐Aldrich, St. Louis, MO, USA), 0.2 mM Iron(III) acetylacetonate (F300, Sigma‐Aldrich, St. Louis, MO, USA), 0.8 mM Hexadecyltrimethylammonium chloride (CTAC), and 0.5 ml oleic acid were mixed with 10 ml oleylamine. The mixture was stirred in an Argon gas atmosphere for 1 hour at room temperature. Afterward, the solution was heated to 350 °C at 5 °C min^−1^. The temperature was kept constant at 350 °C for 3 hours. Subsequently, the reaction mixture was cooled down to room temperature. FePt nanoparticles were precipitated by adding ethanol and centrifuged at 8000 rpm for 3 min. The particles were redispersed in hexane and again precipitated in ethanol, followed by centrifugation. This process was repeated at least five times to ensure the removal of the unreacted species and excess surfactants.

The FePt nanoparticles were coated with PDA to decrease the hydrophobicity of the nanoparticles and create dispersion in the physiological media. First, nanoparticles in DMSO were prepared at a ratio of 1:10 (w/v) and sonicated under 150 W in a beaker for 15 min. Then, the nanoparticles in the TRIS buffer were placed on it at a ratio of 1:10 (w/v), and dopamine hydrochloride (A1113606, Thermo Scientific, Waltham, MA, USA) was added at a ratio of 1:1 (w:w) for dopamine hydrochloride and nanoparticles. After adding all the ingredients, they were mixed in a magnetic stirrer at 1000 rpm for 24 h. The PDA‐coated nanoparticle solution obtained at the end of 24 h was centrifuged at 8000 rpm for 10 min and washed with distilled water three times to eliminate the excess unconjugated PDA.

### Scanning Transmission Electron Microscopy (STEM) with Energy Dispersive X‐Ray Spectroscopy (EDX)

JEOL ARM200F scanning transmission electron microscope (STEM) was used to measure particle size and coating thickness on PDA‐coated FePt nanoparticles. After sonication, 1 µl of nanoparticle suspension was deposited on a copper grid and freeze‐dried (Alpha 1‐2 LSC‐basic, Christ Inst., Hagerstown, MD, USA). The samples were observed under 200kV accelerating voltage, with a maximal 0.5 angstrom resolution. A semi‐automated particle analysis algorithm in ImageJ Fiji (NIH, Bethesda, MD, USA) was used to measure the nanoparticle size distribution of the nanoparticles. JEOL JED‐2300 Analysis Station was used to determine the elemental composition of the PDA‐coated and uncoated FePt nanoparticles, and the EDX analyses were performed according to the kV value of each element.^[^ ^87^ ^]^

### Vibrating Sample Magnetometer (VSM) Analysis

The Microsense EZ7 vibrating sample magnetometer (VSM) was used to study the magnetic properties of the nanoparticles. One milligram of nanoparticles was dispersed with chloroform, dried on the coverslide, and loaded on the 8 mm transverse holder for the measurements. In addition, after manufacturing MDMSs, they were programmed in different directions by applying a 1.8 Tesla uniform magnetic field for magnetically driven helical swimming behavior.

### Infrared, UV, and Visible Light Spectroscopy Measurements

The Spectrum‐2 Fourier Transform Infrared (FTIR) Spectroscope (Perkin Elmer, Waltham, MA, USA) was used to confirm the functional groups like ‐NH2, ‐OH, and ‐COOH in solution to observe sufficient dexamethasone loading in the 3D printing precursors. The FTIR spectroscopy was carried out over a wide wavenumber range of 500‐4000 cm^−1^.

LAMBDA 1050+ UV/Vis Spectrophotometer (Perkin Elmer, Waltham, MA, USA) used for UV‐visible light spectroscopy. The absorption peaks of the samples were measured between 250 and 800 nm using the quartz cuvettes for minimum background scattering and noise.

### Rheological Analyses

Rheological measurements were carried out with a Discovery HR‐3 Hybrid Rheometer (TA Instruments, New Castle, USA) equipped with a 20 mm Smart‐Swap flat hatched plate in both sweep modes with different frequencies and strain levels. The working gap was set to 5 mm, which was the thickness of the hydrogels. The rheometer was calibrated before every measurement session, which validates consistent average thickness for hydrogels for all experiments. Storage and loss moduli profiles were measured under different frequencies (0.1 to 100 rad s^−1^) and strains (0.1% to 10%) in a humid, room‐temperature environment to minimize evaporation. The dynamic rheometer recorded the hydrogel mechanical properties via the storage and loss moduli (G’ and G″).

### Digital Light Processing 3D Printing

Dexamethasone 21‐phosphate (700 mg ml^−1^) (DEX, J64083‐06, Thermo Fisher Scientific, Waltham, MA, USA) was loaded into the PEGDA hydrogel precursor (#024, Advanced Biomatrix, Carlsbad, CA, USA) and nanoparticle solution by using 1‐h vortex‐stirring. To validate the complete loading of the DEX to the hydrogel precursor, high‐performance liquid chromatography, which is explained in detail below, was used, and the initial concentration was found as 700.67±2.74 mg ml^−1^ (n = 3). The volume of each swimmer was calculated as 4.704 mm^3^ from 3D models, and ≈3.29 mg DEX corresponds to the loaded drug amount for each of them. After the complete dissolution of DEX, the magnetic degradable milliscale swimmers (MDMS) were printed with a digital light processing (DLP) 3D bioprinter (Lumen‐X Gen 2, CELLINK, Gothenburg, Sweden) with 30 mW cm^−2^ light intensity, 12 s of exposure, and 4x burn‐in time.^[^ ^89^ ^]^ After printing, the swimmers were developed in the phosphate‐buffered saline (PBS, pH 7.4, Gibco, Waltham, MA, USA) and transferred to PDA‐coated FePt nanoparticle solution (50 mg ml^−1^) in TRIS buffer to coat their surface with 1‐h vortex stirring, and MDMSs were washed with PBS afterward. All 3D models for printing can be found in the Supporting Information.

### Magnetic Actuation

For both medical imaging experiments and magnetic actuation experiments in ocular physiological media, the fresh porcine eyes were purchased from Ulmer Fleisch food factory, Ulm, Germany. Within 6 hours after the euthanasia of the animals, an enucleated eye was stabilized to the holder, and the MDMS were injected with a sterile 17G syringe in the anterior chambers of the porcine eyes before OCT and PA imaging. A sterile, thin‐walled 17G syringe was used because of its small incision size, 1.27 mm. Besides that, aqueous humor was removed from another set of fresh porcine eyes with the help of a sterile 17G syringe. For vitreous collection, a standard microincision vitrectomy procedure was followed, and the loss and storage moduli of the vitreous humor were measured to ensure its mesh integrity (Figure S9, Supporting Information).^[^ ^90^ ^]^ Storage modulus of the vitreous was 10.72 ± 3.84 Pa, and loss modulus of the vitreous was 2.21 ± 1.08 Pa in 1% oscillating strain with 6 rad/s conditions (*n* = 6).

The milliscale swimmers were actuated using a custom‐made three‐dimensionally rotating permanent magnet system, which provides rotating magnetic fields with various frequencies, from 1 to 9 Hz, and an amplitude of 10 mT that enables helical swimming actuation and steering control for various biological media, including vitreous humor and aqueous humor (Figure S10, Supporting Information). For the custom‐made magnetic setup, a 30 mm‐diameter NdFeB N40 permanent magnet (EarthMag GmbH, Dortmund, Germany) was actuated in two axes.^[^ ^67^, ^91^ ^]^ The generated magnetic field was validated with a 3D magnetic sensor (TLE493D‐W2B6, Infineon AG, Neubiberg, Germany) before each experiment. The magnetic actuation videos were collected with a camera‐integrated surgical stereomicroscope (M205C, Leica, Wetzlar, Germany). The image analysis and MDMS tracking in magnetic actuation and biomedical imaging experiments were analyzed with a custom‐made open‐access MATLAB algorithm, which could be found at the “github.com/erdosty/PHOTODOCTOR” website.

### Biomedical Imaging Methods

PA imaging was performed with a Multispectral Photoacoustic Tomography device system with a handheld 3D photoacoustic probe (MSOT 256‐element transducer, iThera Medical, Munich, Germany). The MDMS and the porcine eyes were embedded in an agar phantom prepared with 1.5 g in 100 mL agar in distilled water. The measurements were then taken for a range of wavelengths (660 – 980 nm), and each image was repeated three times for each laser pulse and then averaged. According to these measurements, 800 nm was selected for the best contrast in the image sequences, which were taken at ten frames per second. Then, a volumetric image of 20 × 20 × 20 mm^3^ was constructed from three orthogonal imaging planes.

For OCT imaging, MDMS were injected into the intraocular area with a 17G sterile syringe and observed via Spectral Domain OCT System (TEL320C1, Thorlabs, NJ, USA). The OCT images inside the eye were recorded with an image speed at a medium sensitivity (76 kHz). The refractive index was set to 1.00, and the Hann filter was used for the apodization window. The A‐scan averaging was set to 1, and the B‐scan averaging to 1 with a pixel size of 6.5 µm.

For US imaging, the B‐sectional mode of the Vevo3100 ultrasound imaging system with MX700 transducer was used (FUJIFILM, Tokyo, Japan).

### Accelerated Aging Tests

The milliscale swimmers were incubated at 67 °C with shaking for up to 21 days with daily solution changes. Both hydrolytic and oxidative degradation mechanisms were investigated.^[^ ^50^ ^]^ The time for degradation was calculated and reported in the results, in terms of accelerated aging test (AAT) days, which was calculated according to medical device guidelines by using this equation:^[^ ^57^ ^]^

(1) $$T_{AAT}=T\times 2^{\left(t-t_{ref}\right)/10}$$

In this formula, *T* is the actual number of days, *t_ref_* is the body temperature, and *t* is the temperature at which the AAT was performed. While MDMS were incubated in a 5 mm sodium hydroxide (NaOH) solution for accelerated hydrolytic degradation, MDMS were incubated in a 3% hydrogen peroxide solution with 1.25 mm cobalt chloride for accelerated oxidative degradation.^[^ ^92^ ^]^ For each time point, the MDMS were cleaned from excessive solution and weighed to determine the equilibrium wet mass, and the masses of the MDMS were monitored daily until complete dissolution occurred (XPR2U, Mettler Toledo, OH, USA). All groups were at least triplicated, and the equilibrium mass was normalized at each time point relative to the initial mass.

### Cell Culture and Cell Viability Assays

The differentiated EOC13.31 cells (ATCC Cat# CRL‐2468, RRID: CVCL_5743) were used as the retinal microglial cell model, and the differentiated ARPE‐19 human retinal pigment epithelial cells (ATCC Cat# CRL‐2302, RRID: CVCL_0145) were used as the model of the human retinal pigment epithelium in the cell culture experiments, which were the well‐established cell culture models for the respective cells.^[^ ^62^, ^93^ ^]^ A conditioned 10% fetal bovine serum (FBS) supplemented Dulbecco‐modified Eagle medium (DMEM) with LADMAC cells (ATCC Cat# CRL‐2420, RRID: CVCL_2550) was used for EOC13.31 microglial cell differentiation until further experiments for cytokine measurements and immunofluorescence stainings. Similarly, the ARPE‐19 human retinal pigment epithelial cell line was cultured in DMEM/F12 supplemented with 10% (v/v) FBS and 1% (v/v) penicillin/streptomycin (Gibco, Grand Island, NY, USA). ARPE‐19 cells were maintained in a modified ARPE‐19 differentiation medium,^[^ ^94^ ^]^ which was DMEM/F12 medium supplemented with 1% GlutaMAX(Gibco, Grand Island, NY, USA), 1% FBS, 1% penicillin/streptomycin, 1% non‐essential amino acid solution, 1% N1 neural growth supplement, taurine (0.25 mg ml^−1^), hydrocortisone (20 ng ml^−1^), and 10 mm nicotinamide, until further experiments for drug release, epithelial barrier function tests, and immunofluorescence stainings. All cell lines were incubated contamination‐free from fungi, bacteria, or mycoplasma in a humidified incubator at 37 °C and 5% CO_2_ during the preparation and execution of experiments.

For cell viability tests, calcein‐AM (green) and ethidium homodimer‐1 (red) incorporated LIVE/DEAD assay (L34961, Thermo Fisher Scientific, Waltham, MA, USA) and CellTiter‐Glo (CTG) luciferase assay (G7570, Promega, Madison, WI, USA) were used. The cells were seeded into µ‐Slide eight‐well plates (ibidi GmbH, Gräfelfing, Germany) for LIVE/DEAD assay and 6‐well plates for the CTG assay and incubated with or without MDMS or an equivalent amount of DEX‐supplemented cell media. After incubation, the LIVE/DEAD assay was made according to the manufacturer's recommendation, and the cells in eight‐well plates were observed with a fluorescence microscope (BZ‐X800, Keyence, Osaka, Japan). Also, the CTG assay mixture was added to the 6‐well plates after incubation, and the luminescence signal was measured with a multimodal plate reader (Infinite 200 Pro, Tecan, Mannedorf, Switzerland).

### Drug Release Experiments

Drug release dynamics of the MDMS were measured by seven days of incubation of the MDMS in ARPE‐19 cells seeded in 6‐well plate wells with 1 ml of cell culture medium, which was DMEM/F12 with 10% FBS, until the DEX concentration in the solution was achieved at the equilibrium level. After the incubation period, the cell culture media were collected and filtered with a 0.45 µm syringe filter. For dexamethasone concentration measurements, 1260 Infinity II High‐performance liquid chromatography (HPLC) with ZORBAX Rx‐C8 Column (Agilent Technologies, Santa Clara, CA, USA). In 80% methanol and 20% distilled water mobile phase, 10 µl from each sample was measured at 254 nm wavelength, under 50 °C, with a 0.5 ml min^−1^ flow rate for 10 min. The DEX concentrations for each time point were compared and calculated using HPLC peak values of DEX concentration standards (Figure S8, Supporting Information).

### Epithelial Barrier Function Tests

The barrier function of ARPE‐19 cells was measured using transepithelial electrical resistance (TEER) and fluorescein isothiocyanate (FITC)‐Dextran permeability assay. For TEER measurements, a handheld epithelial voltohmmeter (EVOM2, World Precision Instruments, Sarasota, FL, USA) was used on confluently seeded ARPE‐19 cell layers on 6‐well and 24‐well transwells with 0.4 µm pore size (Sarstedt, Nümbrecht, Germany). When the epithelial barrier formation was observed with TEER measurements, MDMS or DEX were introduced to the transwells, and TEER measurements were continued. All transepithelial electrical resistance values were written as ohm×cm^2^. Similarly, MDMS and DEX were introduced to the ARPE‐19 epithelial layers on transwells for the FITC‐Dextran permeability assay on day 7. After 24 h of incubation, FITC‐Dextran (FD40, average mol wt 40,000, Sigma‐Aldrich, St. Louis, MO, USA) was introduced to the upper chamber of the transwell, and cell media from the lower chamber was collected every hour until equilibrium. The FITC‐Dextran levels in the collected cell culture media were measured with a multimodal plate reader with filters for 485 and 535 nm excitation and emission, respectively.

### Immunofluorescence Staining and Imaging

ARPE‐19 and EOC13.31 cells (2.5 × 10^5^ cells per well) were incubated with/without MDMS or an equivalent amount of DEX for 48 h at 37 °C in a cell culture incubator. After incubation, both cells were fixed with 4% paraformaldehyde and washed three times with phosphate‐buffered saline with 0.1% Triton X‐100 (PBS‐T). Then, cells were blocked in a Superblock solution (37515, Thermo Scientific, Waltham, MA, USA) While ARPE‐19 cells were incubated with the mouse anti‐ZO‐1 (33‐9100, Thermo Fisher) and the rabbit anti‐CRALBP (PA5‐100178, Thermo Fisher), EOC13.31 cells were incubated with the rabbit anti‐Iba1 (MA5‐29012, Thermo Fisher) antibodies for 90 min in 37 °C and washed three times with PBS‐T. Then, the cells were incubated with the goat anti‐mouse recombinant secondary antibody with Alexa Fluor 488 (A28175, Invitrogen) and goat anti‐rabbit cross‐adsorbed secondary antibody with Alexa Fluor 555 (A‐21428, Invitrogen) for 90 min at 37 °C, according to the corresponding host species of the primary antibodies. After antibody incubations, all samples were washed three times with PBS‐T and then mounted with a DAPI‐supplemented mounting medium (ab104139, Abcam) to observe nuclei. Finally, immunofluorescence imaging was done using the Leica TCS SP8 DMi8 confocal laser scanning microscope (Leica, Wetzlar, Germany).

### Measurement of Immunomodulatory Effects

The EOC13.31 cells were incubated for 48 h with or without LPS (2 µg mL^−1^) in combination with DEX or implant. While the cells were fixed for the immunofluorescence stainings mentioned above, the supernatants were collected for the enzyme‐linked immunosorbent assay (ELISA). The concentrations of the inflammation regulatory cytokines, IFN‐γ, IL‐1β, IL‐6, IL‐10, TNF‐α, and G‐CSF, were measured with commercially available kits (SimpleStep ELISA, Abcam, Cambridge, UK) according to the manufacturer's recommended protocols. The cytokine levels for each experimental group were measured blindly in triplicate.

### Statistical Analyses

For statistical analyses, two experimental groups were tested with a t‐test, multiple experimental groups with one‐way ANOVA, and numerous experimental groups with several time points were statistically tested with two‐way ANOVA (Prism 8, GraphPad, San Diego, CA, USA). The *p*‐value < 0.01 was considered statistically significant for the experimental results. Quantitative data in figures were expressed as mean  ±  standard deviation (SD). Each experiment was at least triplicated to confirm replicability.

## Conflict of interest

The authors declare no conflict of interest.

## Author Contributions

E.Y. and M.S. designed the project, proposed the idea, and secured the funding. E.Y. wrote the manuscript with input and corrections from all authors. E.Y. and U.B. designed small‐scale magnetic degradable robots. F.W. and E.Y. produced the magnetic nanoparticles. A.K. designed the magnetic actuation setup. E.Y. and F.W. conducted the PA and the OCT imaging experiments. E.Y., Eray Y., and D.S. conducted the drug release experiments. Eray Y., Y.Y., and E.Y. conducted the cell culture experiments. E.Y. and M.H. drew the figures. M.S. supervised the research. All authors contributed to the discussion of the data and overall results.

## Supporting information



Supporting Information

Supplemental Video 1

Supplemental Video 2

Supplemental Video 3

Supplemental Video 4

Supplemental Video 5

## Data Availability

This scientific project was carried out under the EU Open Science Framework, and the scientific data that was generated is available to the broader scientific community, as outlined below. The authors declare that all data supporting the findings of this study are available within the paper and its supplementary information. The additional data that support the findings of this study can also be found in the project folder at the EDMOND Open Research Data Repository of the Max Planck Society via this link: https://doi.org/10.17617/3.J0N7UW.
