# Supplementary material for: Magnetically Controllable and Degradable Milliscale Swimmers as Intraocular Drug Implants
Source: Adv Sci (Weinh). 2025 Jun 17;12(34):e07569. doi: 10.1002/advs.202507569 (PMC12442668; doi:10.1002/advs.202507569)
Supplement: Supplementary file 1 — Supporting Information [file ADVS-12-e07569-s003.docx]

**Supplementary Materials**

**for**

**Magnetically Controllable and Degradable Milliscale Swimmers as Intraocular Drug Implants**

Erdost Yildiz^1,2,*^, Ugur Bozuyuk^1^, Eray Yildiz^1,3^, Fan Wang^1,4^, Mertcan Han^1,4^, Alp Can Karacakol^1^, Devin Sheehan^1^, Yan Yu^1^, & Metin Sitti^1,5,*^

^1^ Physical Intelligence Department, Max Planck Institute for Intelligent Systems, Stuttgart, Germany

^2^ Koç University Research Center for Translational Medicine (KUTTAM), Koç University, Istanbul, Turkey

^3^ Department of Molecular Biology and Genetics, Izmir Institute of Technology, Izmir, Türkiye

^4^ Institute for Biomedical Engineering, ETH Zurich, Zurich, Switzerland

^5^ College of Engineering, Koc University, Istanbul, Türkiye

*Correspondence to: [yildiz@is.mpg.de](mailto:yildiz@is.mpg.de), [sitti@is.mpg.de](mailto:sitti@is.mpg.de)

**Contents**

Supp. Table 1: Currently available commercial intraocular drug implants

Supp. Table 2: Mechanical strengths, degradation properties, and drug loading capabilities of various hydrogels

Supp. Table 3: Comparison of photopolymerization-based 3D printing methods for biomedical devices

Supp. Figure 1: TEM and STEM-EDX Images of FePt nanoparticles before PDA coating

Supp. Figure 2: Example STEM images of PDA-coated FePt nanoparticles.

Supp. Figure 3: FTIR spectroscopy results for other hydrogel candidates.

Supp. Figure 4: The dimensions of the MDMS compared to a 1 Euro coin.

Supp. Figure 5: The snapshots from rotation and forward helical swimming of the MDMS

Supp. Figure 6: 3D reconstructions of the photoacoustic images

Supp. Figure 7: Brightfield microscopy image of MDMS on the ARPE-19 cell layer

Supp. Figure 8: HPLC standard curve for dexamethasone concentration measurements

Supp. Figure 9: Rheological properties of the porcine vitreous humor

Supp. Figure 10: The waveform and magnitude of the magnetic field at 6 Hz rotation of the permanent magnet.

Supp. Video 1: Actuation of the MDMS in the intraocular fluids

Supp. Video 2: 3D reconstructions for photoacoustic image recordings of the MDMS

Supp. Video 3: Actuation of the MDMS under photoacoustic imaging

Supp. Video 4: Actuation of the MDMS under optical coherence tomography

Supp. Video 5: Actuation of the MDMS under ultrasound imaging

| **Name** | **Degradable** | **Active Ingredient** | **Amount** | **Indications** | **Duration** | **Manufacturer** | **Dimensions** | **References** |
| --- | --- | --- | --- | --- | --- | --- | --- | --- |
| Ozurdex | Yes | Dexamethasone | 700 μg | Macular edema, non-infectious uveitis | 3-6 months | Allergan | 6 mm x 0.46 mm | (Pacella et al., 2013)^[1]^ |
| Durysta | Yes | Bimatoprost | 10 μg | Open-angle glaucoma, ocular hypertension | 4-6 months | Allergan | 1.1 mm x 0.2 mm | (Brubaker et al. 2021)^[2]^ |
| Retisert | No | Fluocinolone acetonide | 0.59 mg | Chronic non-infectious uveitis | 30 months | Bausch & Lomb | 5 x 2 x 1.5 mm | (Jaffe et al., 2006)^[3]^ |
| Vitrasert | No | Ganciclovir | 4.5 mg | CMV retinitis | 5-8 months | Bausch & Lomb | 1 mm x 2.5 mm | (Dhillon et al., 1998)^[4]^ |
| Iluvien | No | Fluocinolone acetonide | 0.19 mg | Diabetic macular edema | 36 months | Alimera Sciences | 3.5 mm x 0.37 mm | (Kane et al., 2008)^[5]^ |
| iDose TR | No | Travoprost | 75 μg | Glaucoma | 6-12 months | Glaukos | 1.8 mm x 0.5 mm | (Szekely et al., 2023)^[6]^ |
| Yutiq | No | Fluocinolone acetonide | 0.18 mg | Chronic non-infectious uveitis | 36 months | EyePoint Pharmaceuticals | 3.5 mm x 0.37 mm | (Testi and Pavesio, 2019)^[7]^ |
| Durasert | Yes | Vorolanib | 440 μg | Age-related macular degeneration | 36 months | EyePoint Pharmaceuticals | 3.5 mm x 0.37 mm | (Patel et al., 2024)^[8]^ |
| I-vation | No | Triamcinolone acetonide | 925 μg | Diabetic macular edema | 18-36 months | SurModics | 0.5 mm x 3 mm (helical coil) | (Dugel et al., 2007)^[9]^ |

**Supplementary Table 1:** Currently available commercial intraocular drug implants

| **Name** | **Maximum Young’s Modulus** | **Hydrolytic Degradation Products** | **Degradation Time** | **Drug Loading Capacity for Glucocorticoids** | **References** |
| --- | --- | --- | --- | --- | --- |
| PLGA | 244 MPa to 2 GPa | Lactic acid and glycolic acid | Approx. 14 days | 7.5 mg/ml | (Chou and Woodrow, 2017)^[10]^, (Wang et al., 2012)^[11]^ |
| PLA | 2.7 GPa | Lactic acid | 84 to 126 days | 1.3 mg/ml | (Gentile et al., 2014)^[12]^, (Farto-Vaamonde et al., 2019)^[13]^ |
| PEG700 | 95.9 kPa | Glycolic acid, acetaldehyde, formaldehyde, and formic acid | 10 hours to 22 days | 80 mg/ml | (Della Sala et al., 2020)^[14]^, (Jain et al., 2017)^[15]^, (Chen et al.,2019)^[16]^ |
| PEGDA | 83 MPa | Poly(ethylene glycol) (PEG) chains, acrylic acid, and carboxylic acid groups | 28 to 56 days | 20 mg/ml | (Filatov et al.,2017)^[17]^,(Freedman et al., 2021)^[18]^, (Stillman et al., 2020)^[19]^, (Mau et al., 2020)^[20]^ |
| Collagen (Type I) | 0.5 to 12 kPa | Hydroxyproline, hydroxylysine, glycine, free amino acids, and peptide chains (3-6 kDa) | 35 to 50 days | 2.0 mg/ml | (Raub et al., 2010)^[21]^, (Leon-Lopez et al., 2019)^[22]^, (Vallecillo et al., 2021)^[23]^, (Xeroudaki et al., 2023)^[24]^ |
| Methacrylated Collagen (Type I) | 1.3 kPa | Collagen fragments, methacrylic acid, hydroxyproline, and hydroxylysine | Approx. 60 days | 1.0 μg/ml | (Gaudet and Shreiber, 2012)^[25]^, (Mostret et al., 2023)^[26]^ |
| Hyaluronic Acid | 40 kPa | Oligosaccharides, disaccharides, and monosaccharides | Approx. 35 days | 0.5 mg/ml | (Heris et al., 2012)^[27]^,(Simulescu et al., 2016)^[28]^, (Zhang et al., 2016)^[29]^ |
| Methacrylated Hyaluronic Acid | 50 kPa | Hyaluronic acid, lactic acid, and kinetic chains of poly(methacrylic acid) | Approx. 56 days | 4.0 mg/ml | (Poldervaart et al., 2017)^[30]^, (Freedman et al. 2021)^[18]^, (Sahoo et al., 2008)^[31]^, (Yang et al., 2025)^[32]^ |
| Gelatine | 33 kPa | Polypeptides, amino acids, incl. glycine, proline, and hydroxyproline | 18 hours | 50 μg/ml | (Tondera et al., 2016)^[33]^, (Taberlet et al., 2017)^[34]^, (Suvarnapathaki et al., 2019)^[35]^, (Javanmardi et al., 2024)^[36]^ |
| Methacrylated Gelatine | 140 kPa | Gelatine backbone fragments, methacrylic acid, glycine, proline, and hydroxyproline | Approx. 56 days | 20 mg/ml | (Rothrauff et al., 2018)^[37]^, (Hutson et al., 2011)^[38]^, (Xiang and Cui, 2021)^[39]^,(Shen et al., 2023)^[40]^ |
| Silk | 14 to 36 GPa | Polypeptides (10-30 kDa), amino acids, particularly glycine, alanine, and serine | Approx. 145 days | 20 mg/ml | (Lepore et al., 2016)^[41]^, (Liu et al., 2015)^[42]^, (Guo et al., 2020)^[43]^, (Akrami et al., 2021)^[44]^ |
| Chitosan | 1.5 to 4.7 GPa | Chito-oligosaccharides, short-chain oligomers, and glucosamine | Approx. 21 days | 0.78 mg/ml | (Aryaei et al., 2012)^[45]^, (Kienzle-Setzler et al., 1982)^[46]^, (EzEldeen et al., 2021)^[47]^, (Khaqan et al., 2019)^[48]^ |
| Methacrylated Chitosan | 12 to 64 kPa | Chito-oligosaccharides, glucosamine monomers, methacrylic acid, and poly(methacrylic acid) fragments | Approx. 224 days | - | (Osi et al., 2021)^[49]^, (Cui et al., 2019)^[50]^, (Bucciarelli et al., 2024)^[51]^ |

**Supplementary Table 2:** Mechanical strengths, degradation properties, and drug loading capabilities of various hydrogels

| **Metric** | **Digital Light Processing (DLP) 3D Printing** | **Stereolithography (SLA) 3D Printing** | **Two-photon Polymerization** |
| --- | --- | --- | --- |
| XY resolution | 35–62 µm | 50–100 µm | 100–200 nm |
| Layer resolution (Z) | 50–100 µm | 25–300 µm | 300–800 nm |
| Minimum feature size | app. 35 µm | app. 50 µm | <100 nm |
| Maximum printable volume | 119 × 67 × 75 mm | 145 × 145 × 185 mm | <1 cm³ |
| Printing speed | 25–30 mm/hour | 40–100 mm/hour | 0.1–1 mm³/hour (voxel volume) |
| Multi-part fabrication efficiency | Parallel polymerization | Serial polymerization | Serial polymerization |
| RMS error for reproducibility | <50 µm | <0.20 mm | <50 nm |
| Approximate fabrication cost per part (EUR) | 5.00 | 5.90 | >500.00 |
| Scalability | High | Moderate | Low |
| References | (Tumbleston et al., 2015)^[52]^, (Yu et al., 2020)^[53]^ | (Chen et al., 2019)^[54]^, (Abbasi et al., 2025)^[55]^ | (Vyatskikh et al., 2018)^[56]^, (Geng et al., 2019)^[57]^ |

**Supplementary Table 3:** Comparison of photopolymerization-based 3D printing methods for biomedical devices


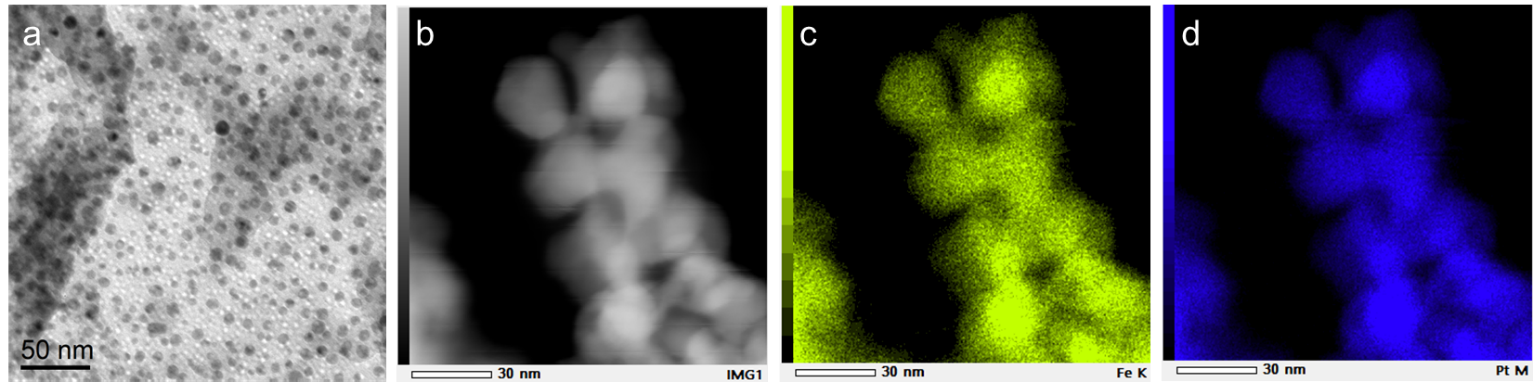


**Supplementary Figure 1:** TEM and STEM-EDX Images of FePt nanoparticles before PDA coating. **(a)** TEM image of FePt nanoparticles before coating. **(b)** STEM image of FePt nanoparticles. Both iron (Fe) **(c)** and platinum (Pt) **(d)** show similar distribution.


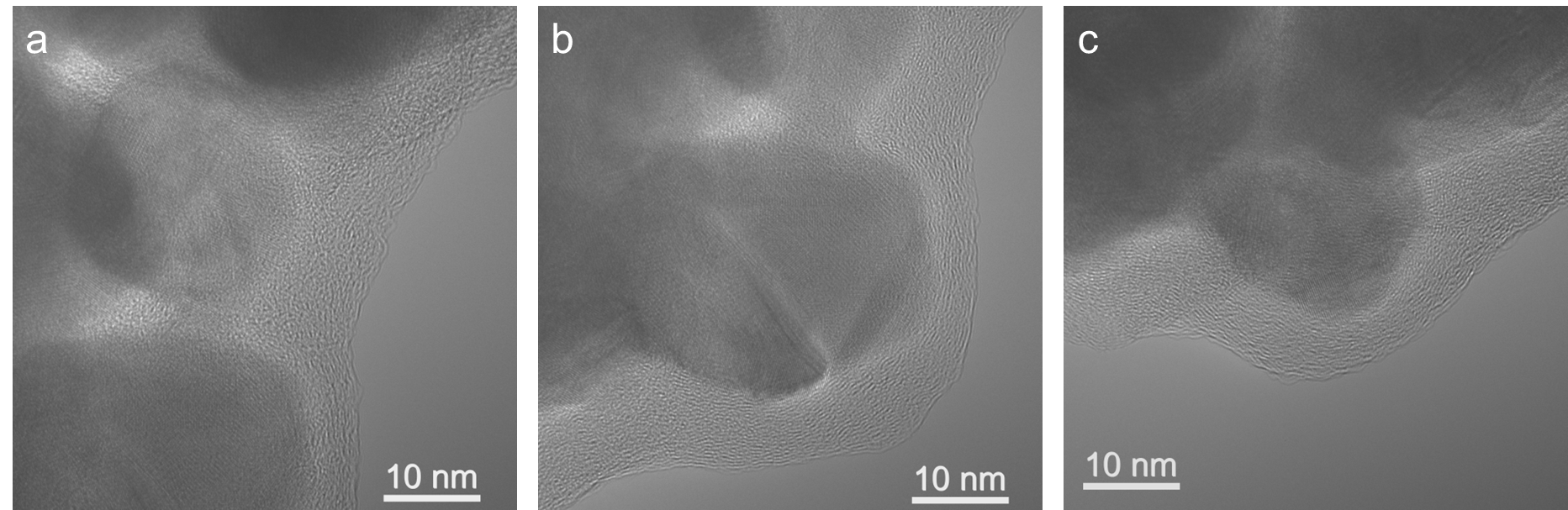


**Supplementary Figure 2:** Example STEM images of PDA-coated FePt nanoparticles.


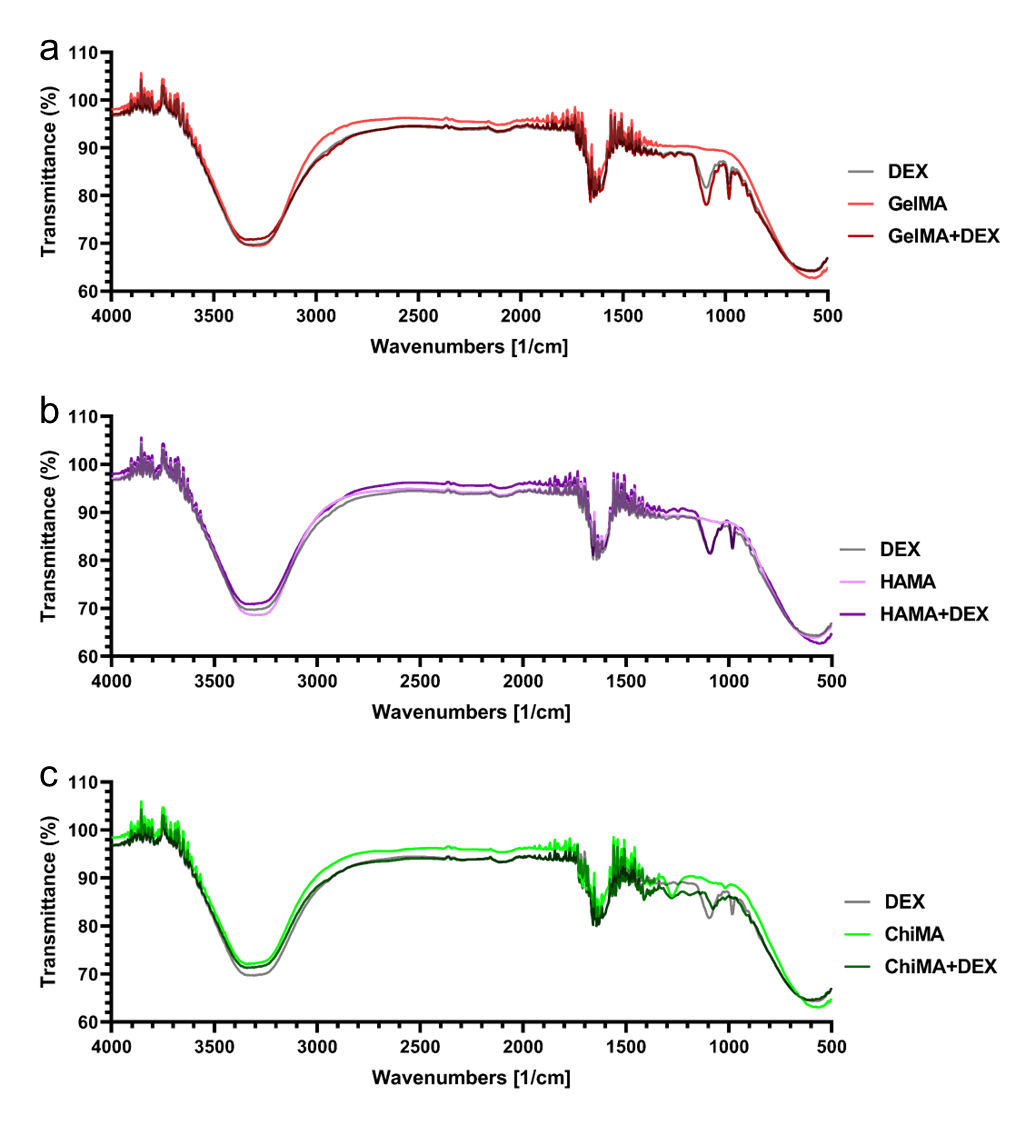


**Supplementary Figure 3:** FTIR spectroscopy results for **(a)** methacrylated gelatin (GelMA), **(b)** methacrylated hyaluronic acid (HAMA), and **(c)** methacrylated chitosan (ChiMA), with and without dexamethasone loading.


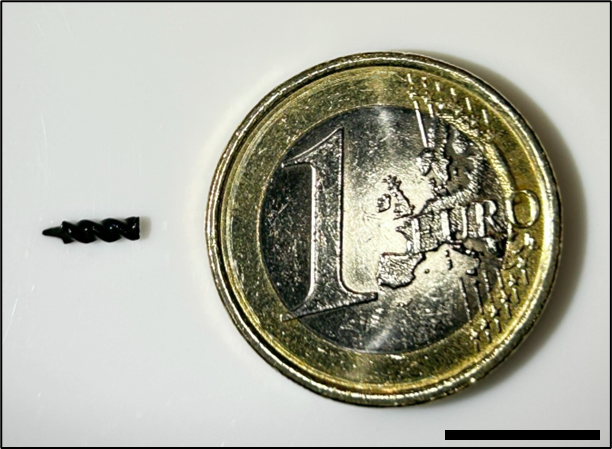


**Supplementary Figure 4:** The dimensions of the MDMS compared to a 1 Euro coin. The scale bar is 10 mm.


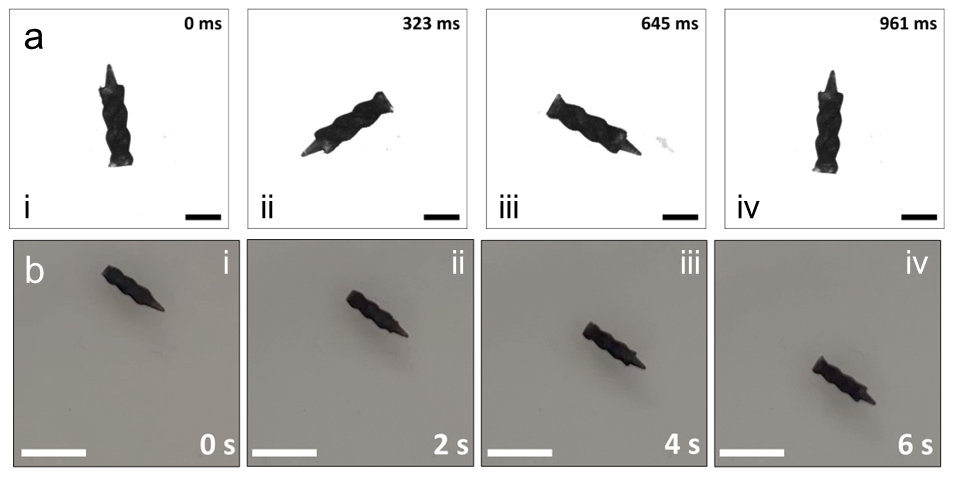


**Supplementary Figure 5:** The snapshots from rotation and forward helical swimming of the MDMS. **(a)** The MDMS yaws on the surface to align with the magnetic axis of the static magnet. The scale bar is 2 mm. **(b)** After the alignment, the magnet starts to rotate, and the MDMS starts to forward helical swimming. The scale bar is 5 mm.


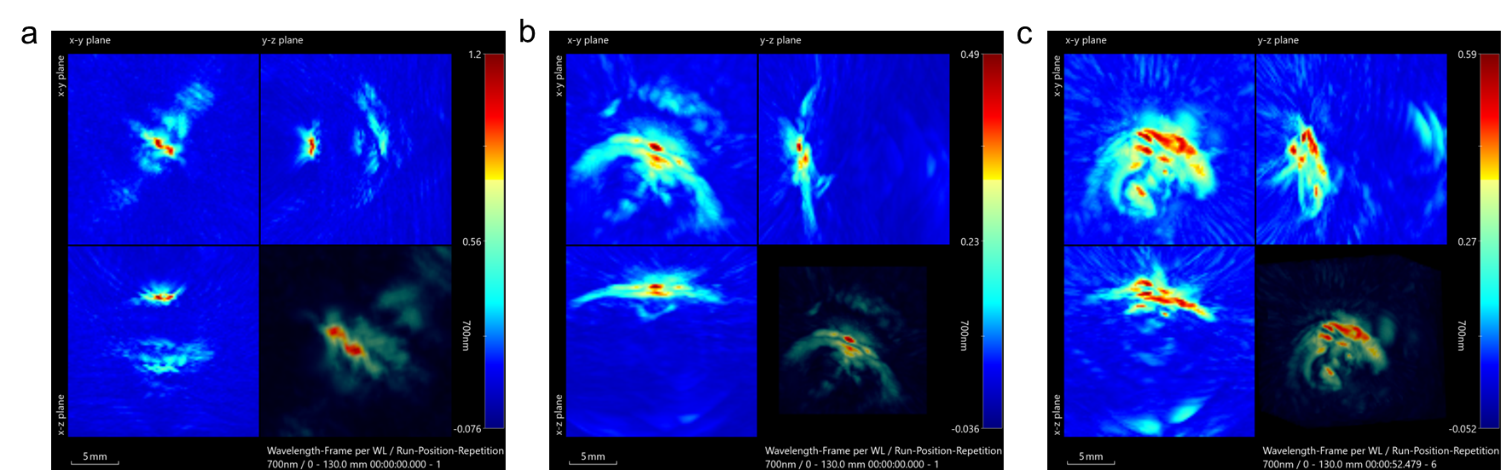


**Supplementary Figure 6:** 3D reconstructions of photoacoustic images. **(a)** The MDMS in the agar, **(b)** the porcine eye before the injection, and **(c)** the porcine eye after the injection of the MDMS.


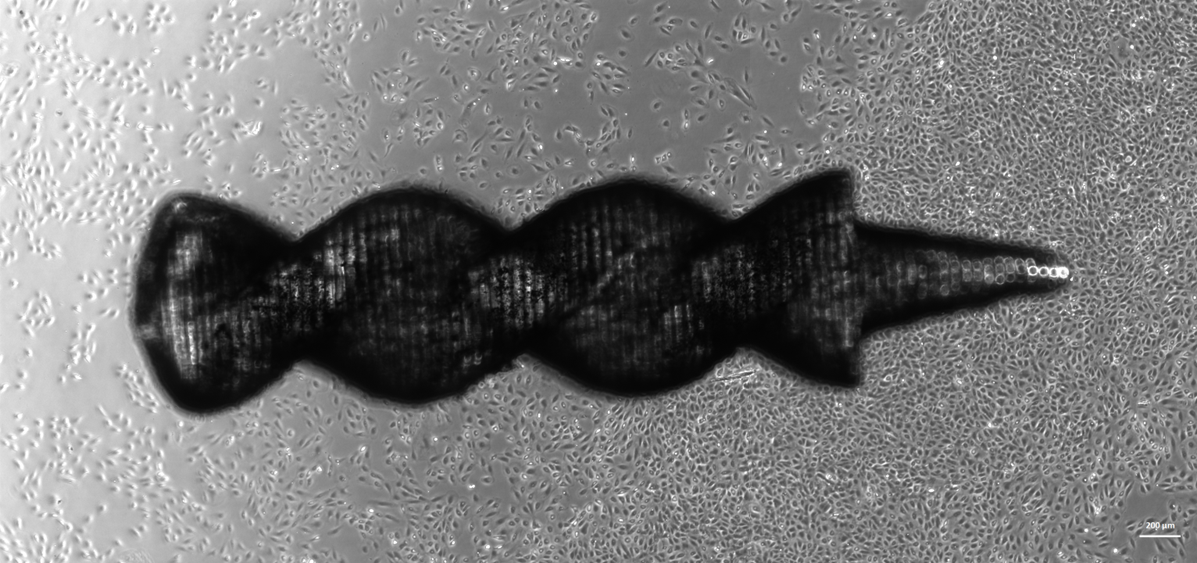


**Supplementary Figure 7:** Phase-contrast microscopy image of the MDMS on the ARPE-19 cell layer. The scale bar is 200 μm.


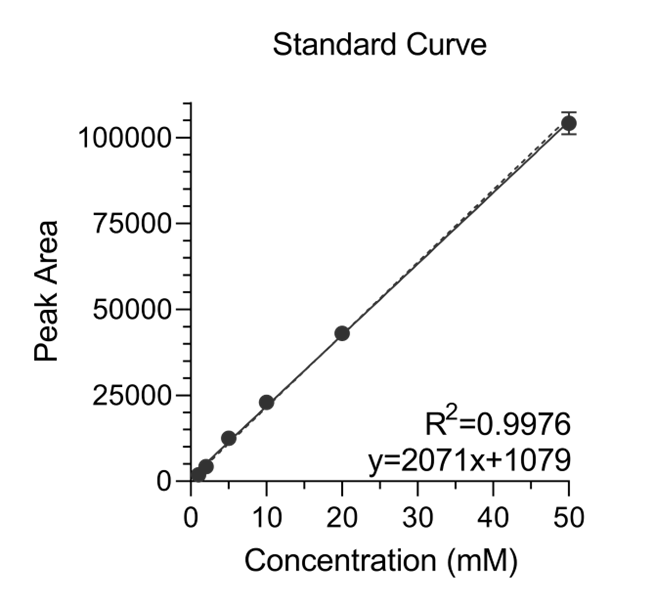


**Supplementary Figure 8:** HPLC peak area-to-concentration standard curve for dexamethasone standard solutions.


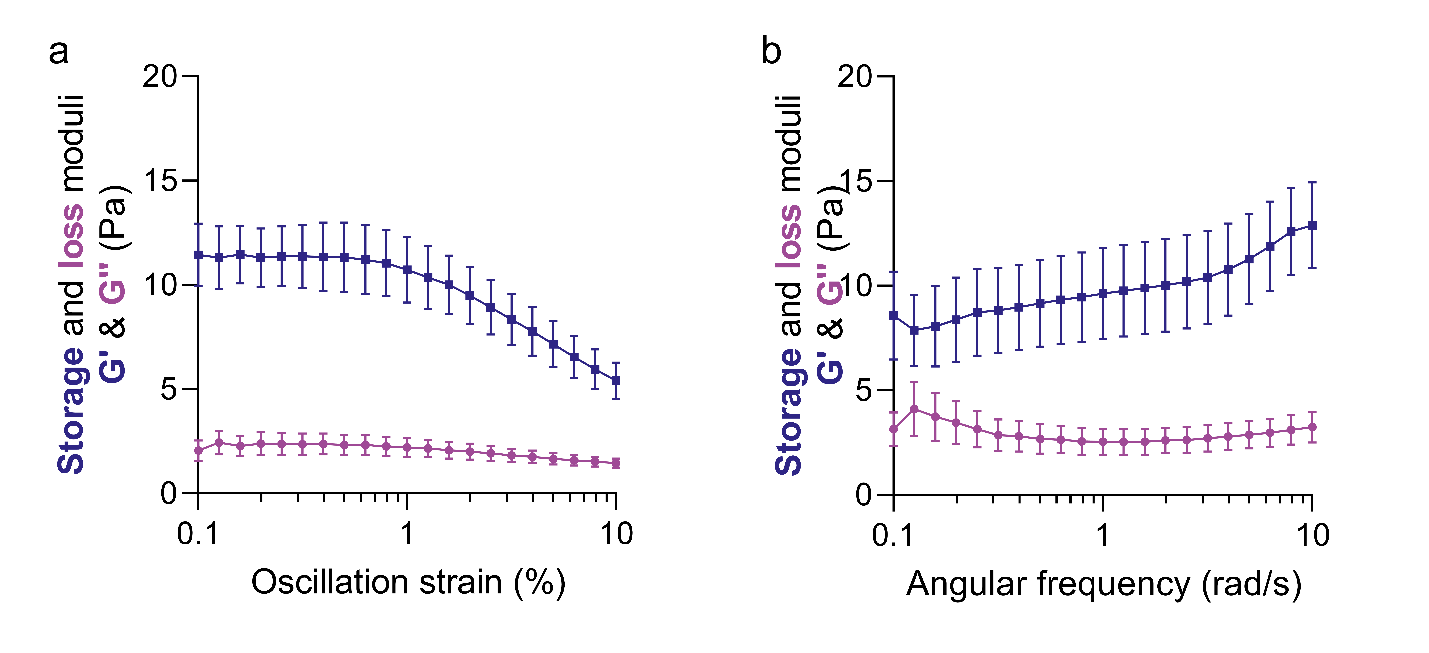


**Supplementary Figure 9:** Rheological properties of the porcine vitreous humor. **(a)** The storage and loss moduli of the vitreous under varying oscillation strain (%). **(b)** The storage and loss moduli of the vitreous under varying angular frequency (rad/s). The plots were drawn with means ± standard errors of the mean.


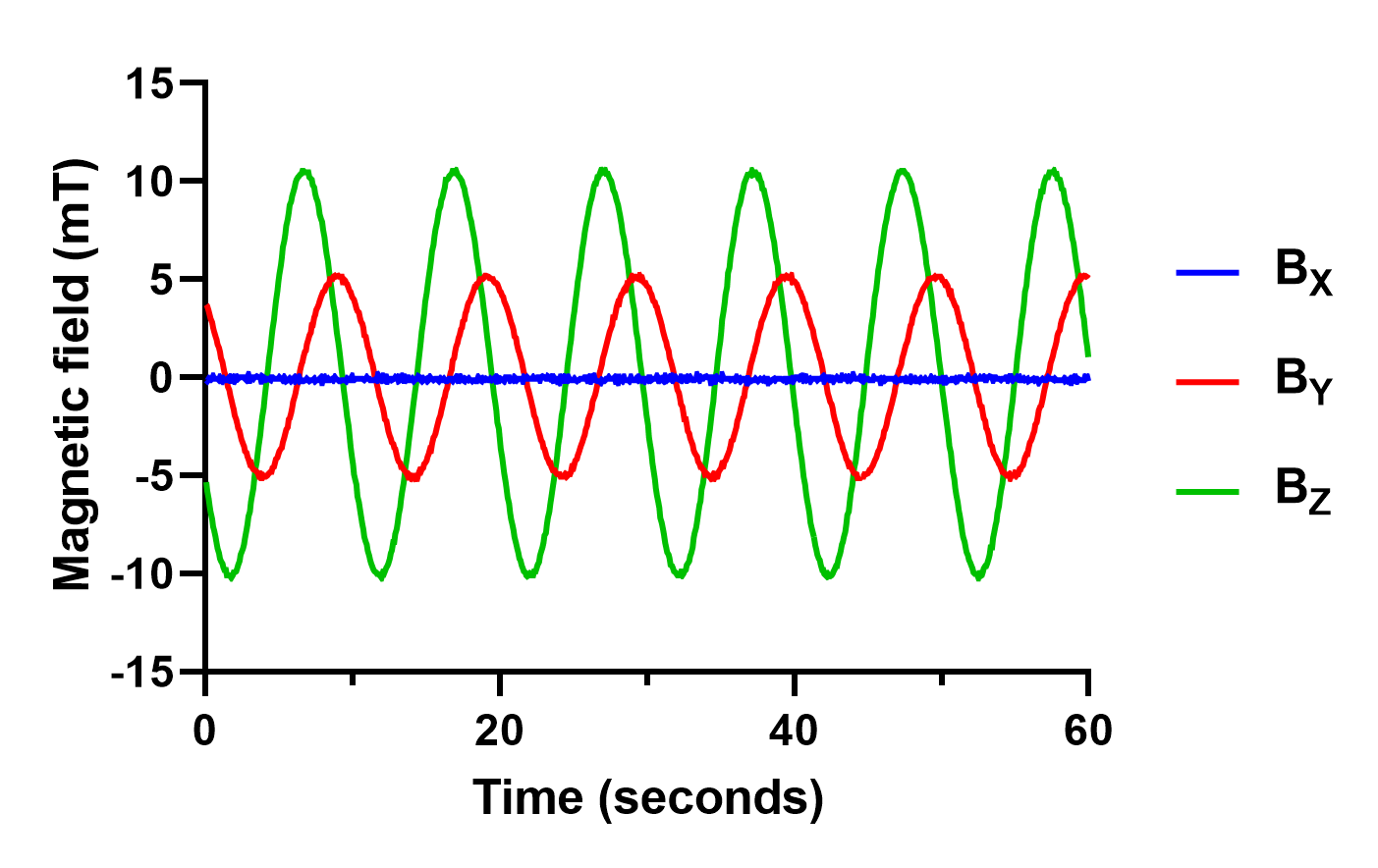


**Supplementary Figure 10:** The waveform and magnitude of the magnetic field at 6 Hz rotation of the permanent magnet.

**References**

[1] E. Pacella, A. R. Vestri, R. Muscella, M. R. Carbotti, M. Castellucci, L. Coi, P. Turchetti, F. Pacella, *Clin. Ophthalmol.* **2013**, *7*, 1423.

[2] J. W. Brubaker, S. L. Mansberger, J. D. Branch, K. Wang, M. R. Robinson, M. Sehi, *Invest. Ophthalmol. Vis. Sci.* **2021**, *62*, 627.

[3] G. J. Jaffe, D. Martin, D. Callanan, P. A. Pearson, B. Levy, T. Comstock, Fluocinolone Acetonide Uveitis Study Group, *Ophthalmology* **2006**, *113*, 1020.

[4] B. Dhillon, A. Kamal, C. Leen, *Int. J. STD AIDS* **1998**, *9*, 227.

[5] F. E. Kane, J. Burdan, A. Cutino, K. E. Green, *Expert Opin. Drug Deliv.* **2008**, *5*, 1039.

[6] G. Szekely, L. J. Katz, L. A. Voskanyan, I. D. Raymond, J.-Y. Yang, L. V. Doan, K. G. Stephens, T. P. Mena, D. S. Capel, S. Placinta, R. J. Wilson, D. N. Armijo, C. L. Davis, D. Applegate, T. Navratil, *Invest. Ophthalmol. Vis. Sci.* **2023**, *64*, 4301.

[7] I. Testi, C. Pavesio, *Ther. Deliv.* **2019**, *10*, 621.

[8] S. Patel, P. P. Storey, M. R. Barakat, V. Hershberger, W. Z. Bridges, D. A. Eichenbaum, D. R. Lally, D. S. Boyer, S. J. Bakri, M. Roy, D. A. Paggiarino, *Ophthalmol. Sci.* **2024**, *4*, 100527.

[9] P. U. Dugel, H. L. Cantrill, D. Eliott, T. Mahmoud, R. Avery, S. E. Varner, *Invest. Ophthalmol. Vis. Sci.* **2007**, *48*, 1413.

[10] S.-F. Chou, K. A. Woodrow, *J. Mech. Behav. Biomed. Mater.* **2017**, *65*, 724.

[11] Y. Wang, S. Vaddiraju, L. Qiang, X. Xu, F. Papadimitrakopoulos, D. J. Burgess, *J. Diabetes Sci. Technol.* **2012**, *6*, 1445.

[12] P. Gentile, V. Chiono, I. Carmagnola, P. V. Hatton, *Int. J. Mol. Sci.* **2014**, *15*, 3640.

[13] X. Farto-Vaamonde, G. Auriemma, R. P. Aquino, A. Concheiro, C. Alvarez-Lorenzo, *Eur. J. Pharm. Biopharm.* **2019**, *141*, 100.

[14] F. Della Sala, M. Biondi, D. Guarnieri, A. Borzacchiello, L. Ambrosio, L. Mayol, *J. Mech. Behav. Biomed. Mater.* **2020**, *110*, 103885.

[15] E. Jain, L. Hill, E. Canning, S. A. Sell, S. P. Zustiak, *J. Mater. Chem. B* **2017**, *5*, 2679.

[16] Y. Chen, J. Gu, J. Liu, L. Tong, F. Shi, X. Wang, X. Wang, D. Yu, H. Wu, *Int. J. Nanomedicine* **2019**, *14*, 4211.

[17] N. A. Filatov, D. V. Nozdriukhin, A. S. Bukatin, *J. Phys. Conf. Ser.* **2017**, *917*, 042024.

[18] B. R. Freedman, O. Uzun, N. M. M. Luna, A. Rock, C. Clifford, E. Stoler, G. Östlund-Sholars, C. Johnson, D. J. Mooney, *Adv. Mater.* **2021**, *33*, 2008553.

[19] Z. Stillman, B. M. Jarai, N. Raman, P. Patel, C. A. Fromen, *Polym. Chem.* **2020**, *11*, 568.

[20] R. Mau, T. Reske, T. Eickner, N. Grabow, H. Seitz, *Curr. Dir. Biomed. Eng.* **2020**, *6*, 406.

[21] C. Raub, A. Putnam, B. Tromberg, S. George, *Acta Biomater.* **2010**, *6*, 4657.

[22] A. León-López, A. Morales-Peñaloza, V. M. Martínez-Juárez, A. Vargas-Torres, D. I. Zeugolis, G. Aguirre-Álvarez, *Molecules* **2019**, *24*, 4031.

[23] C. Vallecillo, M. Toledano-Osorio, M. Vallecillo-Rivas, M. Toledano, R. Osorio, *Polymers* **2021**, *13*, 2633.

[24] M. Xeroudaki, M. Rafat, P. Moustardas, A. Mukwaya, S. Tabe, M. Bellisario, B. Peebo, N. Lagali, *Acta Biomater.* **2023**, *172*, 234.

[25] I. D. Gaudet, D. I. Shreiber, *Biointerphases* **2012**, *7*, 25.

[26] D. Mostert, I. Jorba, B. G. W. Groenen, R. Passier, M.-J. T. H. Goumans, H. A. van Boxtel, N. A. Kurniawan, C. V. C. Bouten, L. Klouda, *iScience* **2023**, *26*, 106423.

[27] H. K. Heris, M. Rahmat, L. Mongeau, *Macromol. Biosci.* **2012**, *12*, 202.

[28] V. Simulescu, M. Kalina, J. Mondek, M. Pekař, *Carbohydr. Polym.* **2016**, *137*, 664.

[29] Z. Zhang, X. Wei, J. Gao, Y. Zhao, Y. Zhao, L. Guo, C. Chen, Z. Duan, P. Li, L. Wei, *Int. J. Mol. Sci.* **2016**, *17*, 411.

[30] M. T. Poldervaart, B. Goversen, M. de Ruijter, A. Abbadessa, F. P. W. Melchels, F. C. Öner, W. J. A. Dhert, T. Vermonden, J. Alblas, *PloS One* **2017**, *12*, e0177628.

[31] S. Sahoo, C. Chung, S. Khetan, J. A. Burdick, *Biomacromolecules* **2008**, *9*, 1088.

[32] F. Yang, D. Lu, Y. Chen, F. Qi, X. Wang, J. Li, Q. Fu, R. Li, D. Wu, J. Wang, D. Liu, L. Zhao, *Int. J. Biol. Macromol.* **2025**, *286*, 137752.

[33] C. Tondera, S. Hauser, A. Krüger-Genge, F. Jung, A. T. Neffe, A. Lendlein, R. Klopfleisch, J. Steinbach, C. Neuber, J. Pietzsch, *Theranostics* **2016**, *6*, 2114.

[34] N. Taberlet, J. Ferrand, É. Camus, L. Lachaud, N. Plihon, *Am. J. Phys.* **2017**, *85*, 908.

[35] S. Suvarnapathaki, M. A. Nguyen, X. Wu, S. P. Nukavarapu, G. Camci-Unal, *RSC Adv.* **2019**, *9*, 13016.

[36] K. Javanmardi, H. Shahbazi, A. Soltani Hekmat, M. Khanmohammadi, A. Goodarzi, *J. Mater. Sci. Mater. Med.* **2024**, *35*, 5.

[37] B. B. Rothrauff, L. Coluccino, R. Gottardi, L. Ceseracciu, S. Scaglione, L. Goldoni, R. S. Tuan, *J. Tissue Eng. Regen. Med.* **2018**, *12*, e159.

[38] C. B. Hutson, J. W. Nichol, H. Aubin, H. Bae, S. Yamanlar, S. Al-Haque, S. T. Koshy, A. Khademhosseini, *Tissue Eng. Part A* **2011**, *17*, 1713.

[39] L. Xiang, W. Cui, *J. Leather Sci. Eng.* **2021**, *3*, 1.

[40] C. Shen, X. Zhao, Z. Ren, B. Yang, X. Wang, A. Hu, J. Hu, *Int. J. Mol. Sci.* **2023**, *24*, 4957.

[41] E. Lepore, M. Isaia, S. Mammola, N. Pugno, *Sci. Rep.* **2016**, *6*, 24699.

[42] B. Liu, Y. Song, L. Jin, Z. Wang, D. Pu, S. Lin, C. Zhou, H. You, Y. Ma, J. Li, L. Yang, K. L. P. Sung, Y. Zhang, *Colloids Surf. B Biointerfaces* **2015**, *131*, 122.

[43] C. Guo, C. Li, D. L. Kaplan, *Biomacromolecules* **2020**, *21*, 1678.

[44] M. Akrami-Hasan-Kohal, M. Eskandari, A. Solouk, *Colloids Surf. B Biointerfaces* **2021**, *205*, 111892.

[45] A. Aryaei, A. H. Jayatissa, A. C. Jayasuriya, *J. Mech. Behav. Biomed. Mater.* **2012**, *5*, 82.

[46] C. A. Kienzle-Sterzer, D. Rodriguez-Sanchez, C. Rha, *Makromol. Chem.* **1982**, *183*, 1353.

[47] M. EzEldeen, J. Loos, Z. Mousavi Nejad, M. Cristaldi, D. Murgia, A. Braem, R. Jacobs, *Eur. Cell. Mater.* **2021**, *41*, 485.

[48] H. A. Khaqan, M. Y. U. Imtiaz, A.-R. H. M. Buksh, *Pak. J. Ophthalmol.* **2019**, *34*.

[49] A. R. Osi, H. Zhang, J. Chen, Y. Zhou, R. Wang, J. Fu, P. Müller-Buschbaum, Q. Zhong, *ACS Appl. Mater. Interfaces* **2021**, *13*, 22902.

[50] Z.-K. Cui, S. Kim, J. J. Baljon, B. M. Wu, T. Aghaloo, M. Lee, *Nat. Commun.* **2019**, *10*, 3523.

[51] A. Bucciarelli, N. Selicato, C. Coricciati, A. Rainer, A. L. Capodilupo, G. Gigli, L. Moroni, A. Polini, F. Gervaso, *J. Mater. Chem. B* **2024**, *12*, 10221.

[52] J. R. Tumbleston, D. Shirvanyants, N. Ermoshkin, R. Janusziewicz, A. R. Johnson, D. Kelly, K. Chen, R. Pinschmidt, J. P. Rolland, A. Ermoshkin, E. T. Samulski, J. M. DeSimone, *Science* **2015**, *347*, 1349.

[53] C. Yu, J. Schimelman, P. Wang, K. L. Miller, X. Ma, S. You, J. Guan, B. Sun, W. Zhu, S. Chen, *Chem. Rev.* **2020**, *120*, 10695.

[54] L. Chen, W.-S. Lin, W. D. Polido, G. J. Eckert, D. Morton, *J. Prosthet. Dent.* **2019**, *122*, 309.

[55] M. Abbasi, P. Váz, J. Silva, P. Martins, *Appl. Sci.* **2025**, *15*, 2245.

[56] A. Vyatskikh, S. Delalande, A. Kudo, X. Zhang, C. M. Portela, J. R. Greer, *Nat. Commun.* **2018**, *9*, 593.

[57] Q. Geng, D. Wang, P. Chen, S.-C. Chen, *Nat. Commun.* **2019**, *10*, 2179.
